# Isolation and Characterization of Secondary Metabolites from *Hydractinia*-Associated Fungus, *Penicillium brevicompactum* MSW10-1, and Their Inhibitory Effects on Hepatic Lipogenesis

**DOI:** 10.3390/md23070275

**Published:** 2025-06-30

**Authors:** Hyeon-Jeong Hwang, Hyeokjin Lim, Jae Sik Yu, Eun Seo Jang, Youngsang Nam, Yeo Jin Lee, Eun La Kim, Seonghwan Hwang, Seoung Rak Lee

**Affiliations:** 1 College of Pharmacy and Research Institute for Drug Development, Pusan National University, Busan 46241, Republic of Korea; 2 Department of Integrative Biological Sciences and Industry, Sejong University, Seoul 05006, Republic of Korea

**Keywords:** *Penicillium brevicompactum*, marine organism-associated fungus, hepatic lipogenesis, metabolic dysfunction-associated steatotic liver disease, lipogenic genes

## Abstract

Marine organism-associated microbes are an important source of structurally diverse and biologically active secondary metabolites exhibiting antimicrobial, anticancer, and anti-inflammatory activities. In this study, we investigated *Penicillium brevicompactum* MSW10-1, isolated from *Hydractinia echinata*, a marine invertebrate adapted to extreme intertidal and subtidal environments with variable temperature, salinity, and oxygen conditions. Through a combination of LC/MS-guided chemical analysis and chromatographic purification, eight secondary metabolites were isolated, including brevicolactones A (**1**) and B (**2**). The absolute chemical structures of **1** and **2** were determined based on NMR spectroscopic experiments, HR-ESIMS data, and quantum chemical ECD calculations. The isolated compounds (**1**–**8**) were evaluated for their ability to inhibit hepatic lipogenesis, a key process in lipid metabolism that is dysregulated in metabolic-dysfunction-associated steatotic liver disease. Furthermore, the inhibitory effects of the isolated compounds on lipid accumulation were further evaluated in primary mouse hepatocytes, using Oil Red O staining. These findings suggested that the isolated compounds may serve as promising candidates for the treatment of metabolic liver diseases associated with lipid dysregulation.

## 1. Introduction

Metabolicdysfunction-associated steatotic liver disease (MASLD), formerly known as non-alcoholic fatty liver disease (NAFLD), is characterized by excessive lipid accumulation in hepatocytes and is strongly associated with obesity, insulin resistance, and dyslipidemia [1,2,3,4,5]. As the global prevalence of MASLD continues to rise, it has become the most common chronic liver disease and a pressing public health concern [6,7]. The liver plays a central role in maintaining systemic energy homeostasis by regulating key metabolic pathways, including de novo lipogenesis, fatty acid oxidation, triglyceride secretion, and uptake [8,9]. The dysregulation of these pathways—particularly the overactivation of the transcription factor SREBP1c and its downstream lipogenic genes FASN and ACC1—leads to abnormal lipid accumulation and impaired liver function [10,11,12,13]. This, in turn, contributes to broader metabolic disturbances, such as insulin resistance and cardiovascular disease. Despite its clinical significance, pharmacological options for MASLD remain limited, underscoring the need for novel therapeutic agents capable of modulating hepatic lipid metabolism [14].

Marine symbiotic microorganisms are recognized as prolific sources of bioactive secondary metabolites with structurally diverse pharmacological properties, making them promising candidates for drug discovery [15]. These organisms have evolved specialized biosynthetic pathways to adapt to unique ecological niches, resulting in the production of metabolites with a wide range of biological activities [16,17]. Unlike free-living microorganisms, marine symbionts exist in close associations with hosts, such as sponges, tunicates, corals, and mollusks, where they engage in complex ecological interactions [18,19,20]. These interactions often drive the biosynthesis of specialized secondary metabolites that play critical roles in host defense, interspecies communication, and environmental competition [21,22,23,24]. Many of these compounds exhibit antimicrobial, anticancer, anti-inflammatory, and neuroprotective properties, underscoring their pharmaceutical potential [25,26]. Among these, marine-derived *Penicillium* species have emerged as valuable sources of structurally diverse and bioactive secondary metabolites [27]. Numerous compounds—including polyketides, alkaloids, and terpenoids—have been isolated from these fungi, demonstrating a wide spectrum of biological activities, such as antibacterial, anticancer, and anti-inflammatory effects [28,29,30]. This metabolic versatility is largely attributed to their adaptation to extreme marine environments, which has driven the evolution of unique biosynthetic machinery [31,32]. In particular, *Penicillium brevicompactum* has been recognized for its production of therapeutically relevant compounds. For instance, phenolic compounds, such as syringic acid, sinapic acid, and acetosyringone, have shown potent antioxidant and cytoprotective effects in HepG2 liver cancer cells [33]. Additionally, the indole alkaloid brevianamide A possesses antibacterial and neuroprotective properties, while the thiosilvatin derivative cis-bis(methylthio)silvatin has been shown to induce apoptosis and inhibit tumor progression in colon cancer cells [34,35]. These findings highlight the remarkable biosynthetic potential of *P. brevicompactum*, reinforcing the importance of further chemical investigation into its secondary metabolites for therapeutic applications.

As part of our ongoing efforts to discover structurally and biologically novel compounds from diverse natural sources [36,37,38,39,40], we explored the bioactive potential of secondary metabolites from *P. brevicompactum* MSW10-1, a fungal strain isolated from the polyp surface of the marine invertebrate *Hydractinia echinata* (Cnidaria). Given the ecological uniqueness of marine-derived fungi and its ability to produce structurally diverse secondary metabolites, we aimed to investigate the metabolic profile of *P. brevicompactum* and identify compounds with potential therapeutic applications. A combination of LC/MS-guided chemical analysis and successive chromatographic purification techniques led to the isolation of eight secondary metabolites (**1**–**8**), including brevicolactones A (**1**) and B (**2**). The structural elucidation of these metabolites was carried out using spectroscopic methods, including NMR (^1^H, ^13^C, ^1^H–^1^H COSY, HSQC, and HMBC) and HR-ESIMS analysis. All the isolated compounds were evaluated for their ability to inhibit hepatic fat accumulation by modulating key lipogenic regulators, such as *SREBP1c*, *FASN*, and *ACC1* in HepG2 cells. Herein, we report the detailed isolation, structural characterization, and biological evaluation of the isolated compounds, highlighting their potential role in regulating hepatic lipid accumulation and their broader significance in natural product-based drug discovery.

## 2. Results

### 2.1. Structural Elucidation of Isolated Compounds

**Figure 1:**
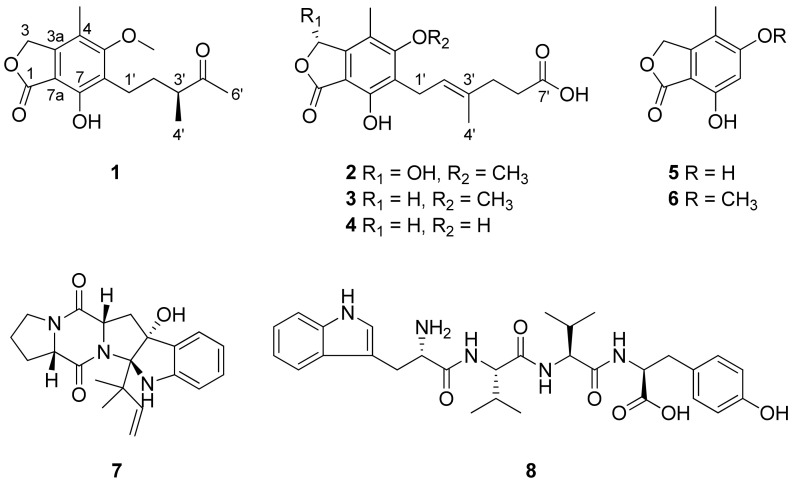
The chemical structures of the isolated compounds **1**–**8**.

Compound **1** (Figure 1) was isolated as an amorphous powder and its HR-ESIMS data (Figure S1) exhibited an ion peak at *m/z* 315.1201 (calcd. for C_16_H_20_O_5_Na, 315.1203), which indicated the molecular formula of **1** was C_16_H_20_O_5_. The ^1^H NMR data (Table 1 and Figure S2) of **1** showed the existence of four methyls [*δ*_H_ 1.13 (3H, d, *J* = 7.0 Hz, H-4′), 2.16 (3H, s, 4-CH_3_), and 2.17 (3H, s, H-6′)], including one methoxy group at *δ*_H_ 3.78 (3H, s, 5-OCH_3_); three methylenes [*δ*_H_ 1.59 (1H, m, H-2′a), 1.92 (1H, m, H-2′b), 2.64 (2H, m, H-1′), and 5.26 (2H, s, H-3)]; and one methine at *δ*_H_ 2.60 (1H, m, H-3′). The ^13^C NMR spectrum (Table 1) of **1**, obtained by the interpretation of its HSQC and HMBC spectra (Figures S4 and S5), revealed a total of 16 carbon resonances, indicating four methyl groups (*δ*_C_ 11.1, 16.4, 27.9, and 61.3), three methylenes (*δ*_C_ 21.9, 33.1, and 70.5), one methine (*δ*_C_ 47.7), six quaternary aromatic carbons (*δ*_C_ 107.6, 117.5, 123.7, 146.7, 154.6, and 164.7), and two carbonyl carbons (*δ*_C_ 173.5 and 215.3). 

**Figure 2:**
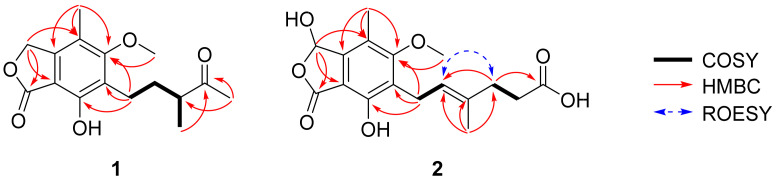
Key ^1^H-^1^H COSY, HMBC, and ROESY correlations of **1** and **2**.

**Figure 3:**
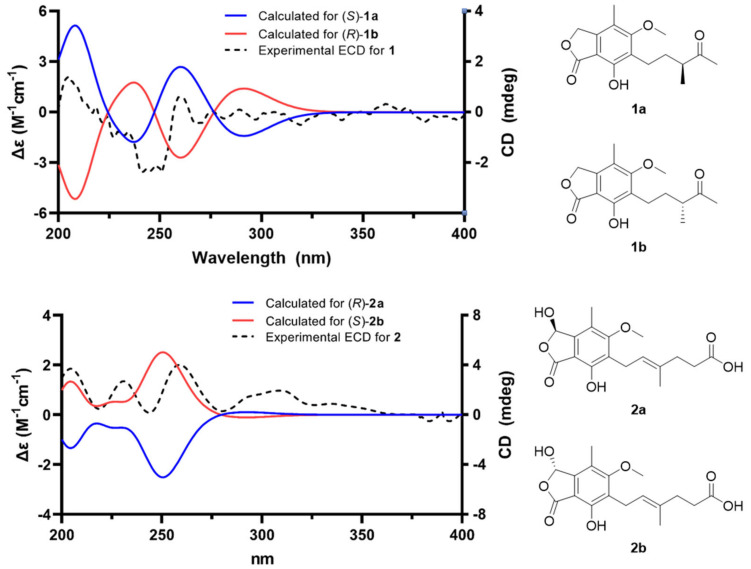
Experimental ECD spectrum of brevicolactones (**1** and **2**) and calculated ECD data of **1a**, **1b**, **2a**, and **2b**.

The HMBC correlations (Figure 2) of **1**, from H_2_-3 to C-1 (*δ*_C_ 173.5), C-3a (*δ*_C_ 146.7), C-4 (*δ*_C_ 117.7), and C-7a (*δ*_C_ 107.6), led to the assignment of the presence of a five-membered lactone ring attached via C-3a and C-7a. Additionally, the existence of skeleton **6** was deduced in its chemical structure based on the HMBC correlations between 4-CH_3_/C-3a, 4-CH_3_/C-4, 4-CH_3_/C-5 (*δ*_C_ 164.7), H_2_-1′/C-5, H_2_-1′/C-6 (*δ*_C_ 123.7), H_2_-1′/C-7 (*δ*_C_ 154.6), and 5-OCH_3_/C-5. An additional six carbons were indicative of 3-methylpentan-2-one, indicated by the analysis of ^1^H-^1^H COSY correlations from H_2_-1′ to H_3_-4′ and key HMBC correlations between H_3_-4′/C-5′, H_3_-6′/C-3′, and H_3_-6′/C-4′ (Figure S3). The position of the 3-methylpentan-2-one was established by HMBC correlations from H_2_-1′ and H_2_-2′ to C-6. After assigning the planar structure of **1**, its experimental electro circular dichroism (ECD) spectrum (Figure 3 and Table S1) was compared with the computed ECD data of two possible isomers (**1a** and **1b**). The *S*-oriented configuration of the methyl group attached at C-3′ of **1** was same as that of euparvic acid, previously identified from *Eupenicillium parvum* [41]. The assigned stereochemistry at C-3′ was further confirmed by a comparison of the experimental (–21.6) and the calculated (–23.1) optical rotation values for **1**. Therefore, the chemical structure of **1**, with its absolute configuration, was determined as shown in Figure 1. According to the previous work, compound **1** was obtained from the fermentation of dihydromycophenolic acid by *Helminthosporium bicolor* and *Aspergillus* sp. [42], but its absolute configuration was determined for the first time in our study. In fact, the absolute chemical structure of **1** was elucidated as depicted in Figure 1 and was trivially named brevicolactone A. 

The HR-ESIMS spectrum (Figure S6) of **2** (brevicolactone B), purified as an amorphous powder, displayed an ion peak at *m/z* 359.1107 (calcd. for C_17_H_20_O_7_Na, 359.1101), corresponding to the molecular formula of C_17_H_20_O_7_. The ^1^H NMR data (Table 1 and Figure S7) of **2** exhibited the presence of three methyls [*δ*_H_ 1.83 (3H, s, H-4′) and 2.27 (3H, s, 4-CH_3_)], including one methoxy group at *δ*_H_ 3.78 (3H, s, 5-OCH_3_); three methylenes [*δ*_H_ 2.28 (2H, m, H-5′), 2.37 (2H, m, H-6′), and 3.42 (2H, d, *J* = 7.0 Hz, H-1′)]; and two methines [*δ*_H_ 6.37 (1H, s, H-3)], including one olefinic proton [*δ*_H_ 5.26 (1H, t, *J* = 7.0 Hz, H-2′)]. The ^13^C NMR spectrum (Table 1 and Figures S9 and S10) of **2** showed three methyls (*δ*_C_ 9.54, 14.7, and 60.0), three methylenes (*δ*_C_ 22.2, 32.3, and 34.2), and two methines (*δ*_C_ 103.0 and 122.7), as well as seven quaternary carbon (*δ*_C_ 107.2, 118.8, 123.9, 133.7, 144.3, 152.9, and 163.7) and two carbonyl carbon (*δ*_C_ 169.5 and 175.9) signals.

A detailed analysis of the spectroscopic data for **2** revealed that its chemical structure closely resembled that of **3**, previously identified as mycophenolic acid from *P. brevicompactum* F01-1358. However, a key difference was observed; the presence of an additional hydroxyl group at C-3 (*δ*_C_ 103.0) in **2**. This was confirmed by key HMBC correlations (Figure 2) of H-3 (*δ*_H_ 6.37) to C-1 (*δ*_C_ 169.5), C-4 (*δ*_C_ 118.8), and C-7a (*δ*_C_ 107.2), clearly establishing the hydroxyl substitution at C-3. A diagnostic ROESY cross-peak between H-2′ and H-5′ was observed, supporting the assignment of an *E*-configuration for the double bond between C-2′ and C-3′ (Figure S11). To determine the absolute configuration at C-3, we performed the calculation of the ECD spectrum (Figure 3 and Table S2) with two possible isomers (**2a** and **2b**). A comparison of the experimental and calculated ECD spectra of the enantiomers confirmed the C-3 configuration of **2** as the *S*-form, as depicted in Figure 1. A comparison of the experimental (+47.8) and the calculated (+18.7) optical rotation values for **2** supported the assignment of the absolute configuration at C-3 as the *S*-form.

The known compounds isolated from *P. brevicompactum* were identified as mycophenolic acid (**3**) [43], O-desmethyl mycophenolic acid (**4**) [44], 5,7-dihydroxy-4-methylphthalide (**5**) [45], 7-hydroxy-5-methoxy-4-methyl-1(3*H*)-isobenzofuranone (**6**) [43], brevianamide E (**7**) [46], and L-tryptophyl-L-valyl-L-valyl-L-tyrosine (**8**) [47] upon comparison with spectroscopic data from previously reported studies (Figures S12–S17). To our knowledge, compound **8** was isolated and structurally identified from *P. brevicompactum* for the first time.

### 2.2. Computational Bioactivity Prediction of Isolated Compounds from P. brevicompactum MSW10-1

To anticipate the biological activities of isolated compounds (**1**–**8**) from *P. brevicompactum* MSW10-1, we performed computational protein target prediction based on their chemical structures (Figure S18). An analysis using the Elsevier Pathway Collection identified three major biological pathways: (1) vitamin A (Retinol) metabolism and the visual cycle, (2) proteins involved in NAFLD, and (3) proteins involved in Tangier disease (Figure S19). Among these, the NAFLD-related proteins were of particular interest, given the increasing prevalence of metabolic disorders and the urgent need for novel therapeutic strategies targeting hepatic lipid accumulation. Notably, pathways (1) and (3) are also linked to lipid metabolism. Vitamin A metabolism regulates lipid homeostasis via retinoic acid signaling, which influences RXR and PPAR activity in lipid storage and breakdown [48,49]. Similarly, Tangier disease involves ABCA1 dysfunction, impairing cholesterol efflux and HDL formation, contributing to lipid imbalances [50,51]. Given these connections, the identified pathways suggested that the isolated compounds identified from *P. brevicompactum* MSW10-1 may broadly influence lipid metabolism.

### 2.3. Effects of All Isolated Compounds on HepG2 Cell Viability

**Figure 4:**
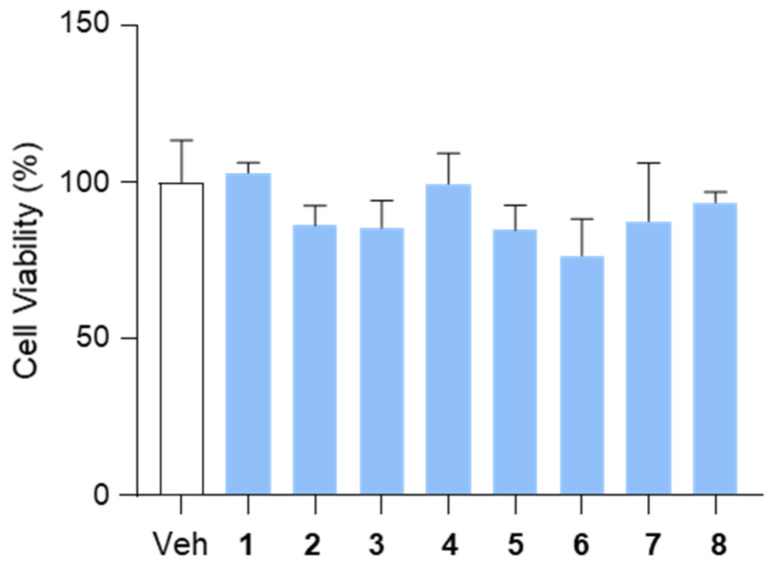
The effects of the isolated compounds (**1**–**8**) on cell viability. HepG2 cells were treated with compounds (10 μM) for 12 h and subjected to a CCK-8 assay to assess cell viability. DMSO (0.1%) served as the vehicle control. Data are presented as means ± SEM. Veh, vehicle.

To assess the effects of the isolated compounds (**1**–**8**) on cell viability, we performed a CCK-8 assay in HepG2 cells. The cells were treated with each compound at a concentration of 10 μM for 12 h, and cell viability was subsequently measured. The results indicated that none of the tested compounds exhibited cytotoxic effects under these conditions, suggesting their suitability for further biological evaluation (Figure 4).

### 2.4. The Inhibition of Lipogenic Gene Expression by the Isolated Compounds

**Figure 5:**
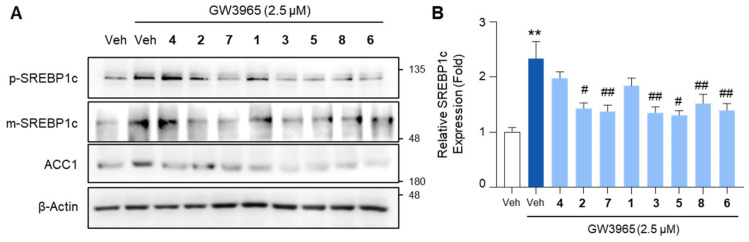
The inhibitory effects of the isolated compounds on the expression of SREBP1c and ACC1. HepG2 cells were treated with GW3965 (2.5 μM) for 12 h, followed by a 30 min pretreatment with the compounds (10 μM). DMSO (0.1%) served as the vehicle control. (**A**) Total cell lysates were subjected to the immunoblot analysis of SREBP1c and ACC1. β-Actin was used as a loading control. (**B**) Relative SREBP1c expression normalized to the expression of β-Actin. Values represent means ± SEM. The statistical evaluation was performed by a one-way ANOVA with a Tukey’s post hoc test for multiple comparisons (** *p* < 0.01 versus vehicle control; ^#^ *p* < 0.05 and ^##^ *p* < 0.01 versus GW3965-treated condition). Veh, vehicle; p-SREBP1c, the premature form of SERBP1c; m-SREBP1c, the mature form of SREBP1c.

Next, we investigated whether the compounds could inhibit the expression of lipogenic genes in hepatocytes. To induce lipogenic gene expression, HepG2 cells were treated with GW3965, a selective agonist of liver X receptor (LXR). LXR is a nuclear receptor that transactivates a multitude of lipogenic factors, including sterol regulatory element binding protein-1c (SREBP1c), fatty acid synthase (FASN), acetyl CoA carboxylase-1 (ACC1), and stearoyl CoA desaturase-1 (SCD1) [52,53]. Among these, SREBP1c is a transcription factor that regulates the transcription of *FASN*, *ACC1*, and *SCD1*. Initially synthesized as a precursor form (p-SREBP1c) in the endoplasmic reticulum membrane, SREBP1c undergoes proteolytic cleavage in the Golgi to generate its mature form (m-SREBP1c), which subsequently translocates to the nucleus to transactivate lipogenic target genes [54]. GW3965 treatment increased the expression of m-SREBP1c in HepG2 cells, conferring the ability to promote lipogenic gene expression, which was attenuated by a pretreatment with the compounds (Figure 5). The compound treatment similarly inhibited the expression of the lipogenic factor ACC1, which is a downstream target gene of both LXR and SREBP1c (Figure 5A). Taken together, these data indicate that the compounds inhibit SERBP1c, the lipogenic transcription factor, induced by LXR activation.

### 2.5. The Effects of the Compounds on Lipogenic Gene mRNA Levels

**Figure 6:**
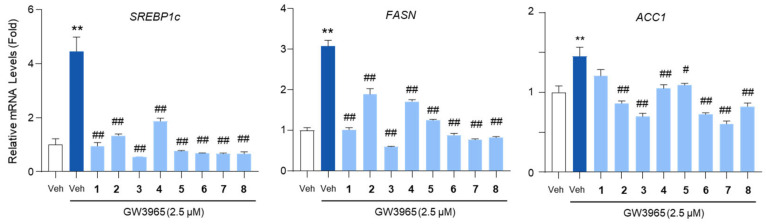
The inhibition of the lipogenic gene expression by the isolated compounds. HepG2 cells were treated with GW3965 (2.5 μM) for 12 h, followed by a 30 min pretreatment with the compounds (10 μM). DMSO (0.1%) served as the vehicle control. The RNA extracted from the cells was subjected to an RT-qPCR analysis of *SREBP1c*, *FASN*, and *ACC1*. *36B4* was used as a reference gene. Values represent means ± SEM. The statistical evaluation was performed by a one-way ANOVA with a Tukey’s post hoc test for multiple comparisons (** *p* < 0.01 versus vehicle control; ^#^ *p* < 0.05 and ^##^ *p* < 0.01 versus GW3965-treated condition).

To further evaluate the impact of the compounds on lipogenic gene expression, we analyzed the mRNA levels of *SREBP1c*, *FASN*, and *ACC1*. Treatment with the compounds markedly reduced the mRNA levels of these genes, with **3** and **7** exerting the most pronounced inhibitory effects (Figure 6). The inhibitory effect of 3 and 7 on SREBP1c mRNA expression was comparable to that of metformin, which suppresses SREBP1c through AMPK activation (Figure S20).

### 2.6. Suppression of Lipid Accumulation in Hepatocytes

**Figure 7:**
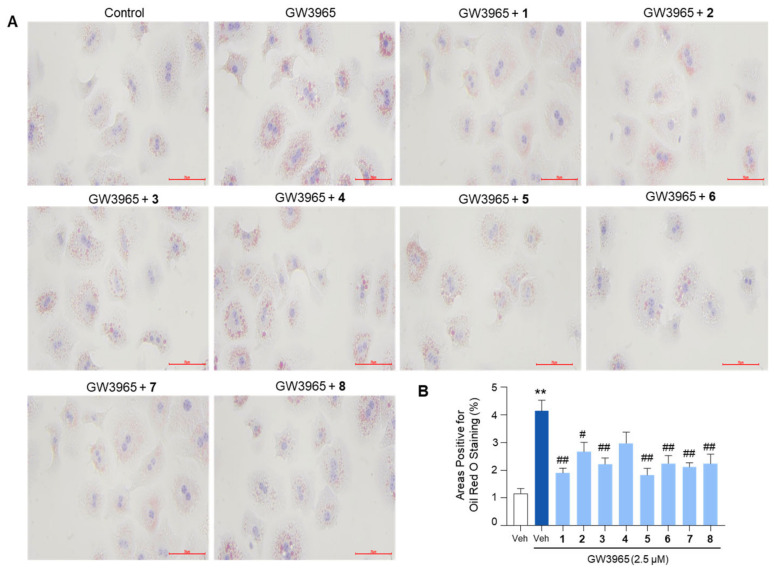
The inhibition of lipid accumulation in hepatocytes by the isolated compounds. Primary mouse hepatocytes were treated with GW3965 (2.5 μM) for 24 h, followed by a 30-min pretreatment with the compounds (10 μM). DMSO (0.1%) served as the vehicle control. (**A**) The cells were subjected to Oil Red O staining for the visualization of intracellular lipid accumulation. Scale bars indicate 70 μm. (**B**) The areas positive for Oil Red O staining were quantified. Values represent means ± SEM. The statistical evaluation was performed by a one-way ANOVA with a Tukey’s post hoc test for multiple comparisons (** *p* < 0.01 versus vehicle control; ^#^ *p* < 0.05 and ^##^ *p* < 0.01 versus GW3965-treated condition).

Given that the compounds inhibited the expression of lipogenic genes, we next investigated whether they could consistently reduce intracellular lipid levels. Oil Red O staining revealed that GW3965 treatment significantly increased lipid accumulation in primary mouse hepatocytes, which was inhibited by pretreatment with the compounds (Figure 7). Overall, these results indicated that the isolated compounds inhibit the expression of lipogenic genes, thereby leading to a reduction in lipid accumulation in hepatocytes.

## 3. Discussion

In this study, we isolated and characterized eight secondary metabolites from *P. brevicompactum* MSW10-1, including brevicolactones A (**1**) and B (**2**), and evaluated their effects on hepatic lipogenesis and lipid accumulation in HepG2 cells. The structural elucidation of **1** and **2** revealed their close relationship to mycophenolic acid, a well-known metabolite of *P. brevicompactum*, but with additional modifications, such as the presence of a hydroxyl group at C-3 in **2**. To evaluate the potential cytotoxicity of the isolated compounds, we first performed a CCK-8 assay in HepG2 cells. None of the compounds exhibited cytotoxicity at a concentration of 10 μM following a 12-hour treatment, confirming their suitability for further biological evaluation.

We then examined whether these compounds could suppress the expression of key lipogenic genes. The HepG2 cells were treated with GW3965, an LXR agonist known to induce SREBP1c-mediated lipogenic gene expression. Immunoblot and RT-qPCR analyses revealed that treatment with the isolated compounds effectively reduced the expression of key lipogenic genes, including *SREBP1c*, *FASN*, and *ACC1*, in HepG2 cells. SREBP1c is a master transcription factor that regulates de novo lipogenesis by promoting the transcription of its downstream target genes, such as *FASN* and *ACC1*. *FASN* encodes fatty acid synthase, a key enzyme responsible for catalyzing the synthesis of long-chain fatty acids, while *ACC1* plays a crucial role in converting acetyl-CoA to malonyl-CoA, the first committed step in fatty acid biosynthesis [55]. These genes are transcriptionally regulated by LXR, an important nuclear receptor involved in lipid metabolism and homeostasis. Although our findings demonstrate that the compounds suppress SREBP1c and its target genes, it remains unclear whether their anti-lipogenic effects act upstream at the level of LXR or directly on SREBP1c. Further studies are needed to elucidate the precise molecular target through which these compounds inhibit lipogenesis. The inhibition of these lipogenic genes suggests that the compounds may act as potential therapeutic agents for MASLD by reducing hepatic lipid accumulation and preventing excessive lipid synthesis in hepatocytes [56].

Furthermore, the isolated compounds were found to suppress lipid accumulation in primary mouse hepatocytes, as evidenced by Oil Red O staining. This finding aligns with the observed downregulation of lipogenic genes, indicating that the compounds not only inhibit gene expression but also reduce the actual lipid content in hepatocytes. This dual effect on gene expression and lipid accumulation highlights the therapeutic potential of these compounds in addressing metabolic liver diseases.

To better understand the basis of their bioactivity, we analyzed the structure–activity relationships of the isolated compounds. The structure–activity relationship (SAR) analysis of the isolated compounds revealed that **3** and **7** showed the most potent anti-lipogenic effects, suggesting a role for specific structural features. Compound **3**, a polyketide with a conjugated carboxylic acid side chain, may exert its activity via enhanced interaction with lipid-regulating pathways. Compound **7**, bearing a rigid indole-fused diketopiperazine core, likely benefits from its conformational stability and cellular permeability. In contrast, compounds **1** and **2**, despite their novelty, exhibited moderate activity, possibly due to their polar hydroxylated phthalide cores. Simpler scaffolds (**5**, **6**) and peptide-like compound **8** showed weaker effects, potentially due to limited lipophilicity or steric hindrance. These findings suggest that lipophilic substituents and fused aromatic systems may enhance biological activity against hepatic lipogenesis.

## 4. Materials and Methods

### 4.1. Chemicals

All the organic solvents used in this study were of analytical grade and purchased from DAEJUNG Chemical Co., Ltd. (Gyeonggi-do, Korea). For the NMR experiments, solvents were obtained from Cambridge Isotope Laboratories (Tewksbury, MA, USA). Fisher Scientific (Ottawa, ON, Canada) provided LC-MS grade methanol, water (purity ≥ 98.0%) and formic acid (purity ≥ 98.0%) for the LC/MS analysis.

### 4.2. General Experimental Procedures

The optical rotation values were determined using a Jasco P-1020 polarimeter (Jasco, Easton, MD, USA). Mass spectrometry analyses, including electrospray ionization (ESI) and high-resolution electrospray ionization mass spectra (HR-ESI-MS), were performed on a Waters Micromass Q-Tof Ultima ESI-TOF mass spectrometer (Waters, New York, NY, USA). Nuclear magnetic resonance (NMR) experiments, such as ^1^H-^1^H COSY, HSQC, and HMBC, were conducted using a Varian UNITY INOVA 800 NMR spectrometer, operating at 800 MHz for ^1^H and 200 MHz for ^13^C. All chemical shifts are reported in parts per million (ppm, *δ*). For preparative separations, a Waters 1525 Binary HPLC pump combined with a Waters 996 Photodiode Array Detector (Waters Corporation, Milford, CT, USA) was employed. Semi-preparative HPLC was carried out using a Shimadzu Prominence HPLC System equipped with SPD-20A/20AV Series Prominence HPLC UV-Vis Detectors (Shimadzu, Tokyo, Japan). LC/MS analyses were performed on an Agilent 1200 Series HPLC system (Agilent Technologies, Santa Clara, CA, USA), which included a diode array detector and a 6130 Series ESI mass spectrometer. An analytical Kinetex column (4.6 × 100 mm, 3.5 μm) was used for these analyses. Thin-layer chromatography (TLC) was performed on Merck precoated silica gel F254 plates and RP-18 F254s plates. The visualization of TLC spots was accomplished under UV light or by heating after spraying with an anisaldehyde–sulfuric acid reagent.

### 4.3. Fungal Material

*P. brevicompactum* MSW10-1 was kindly provided by Prof. Dr. Christine Beemelmanns (Saarland University) and used throughout this study. Polyps of *H. echinata*, obtained from the Alfred Wegener Institute in Sylt, Germany, were aseptically homogenized. The homogenate was diluted with sterile-filtered seawater and plated onto potato dextrose agar (PDA) supplemented with 50 mg/L streptomycin sulfate. The plates were incubated at room temperature for 1–3 weeks. Individual colonies were isolated and repeatedly subcultured onto fresh agar plates to obtain pure cultures. Fungal isolates were grown on PDA at 25 °C in the dark for up to 7 days and preserved as 50% (*v*/*v*) glycerol suspensions at −80 °C. Genomic DNA was extracted to confirm the identity of MSW10-1, and the ITS gene sequence was analyzed. A BLAST search of the ITS gene sequence of MSW10-1 (GenBank accession number MH482922) revealed a 99.81% sequence identity with *P. brevicompactum* B5 (KY921936), confirming the strain as *P. brevicompactum*.

### 4.4. Extraction and Isolation

Overall, approximately 100 PDA plates were inoculated with a 100 µL aliquot of a turbid fungal spore suspension of MSW10-1 in sterile PDB (potato dextrose broth). The suspension was evenly distributed over the agar surface, and the plates were incubated at 25 °C in the dark for up to 10 days. These culture conditions were established based on previous studies indicating that *P. brevicompactum* favors secondary metabolite biosynthesis under dark conditions, with major metabolites, such as mycophenolic acid, typically beginning to be produced around day 3 of cultivation [57,58]. After a period of time, the agar was cut into squares, consolidated, and extracted with 100% methanol (MeOH) overnight. The MeOH solvent was filtered and evaporated in vacuo to acquire a crude MeOH extract (5.1 g). The extract was dissolved in distilled water (700 mL) and successively partitioned with ethyl acetate (EtOAc), yielding 0.42 g of an EtOAc-soluble fraction. The EtOAc fraction was fractionated by preparative reverse-phase HPLC (YMC-Triart C18, 250 × 20 mm, 5 μm, at a flow rate of 5 mL/min) with a gradient solvent system of MeOH-H_2_O (3:7 to 1:0, *v*/*v*) to afford four subfractions (A–D). Compound **5** (1.6 mg, *t*_R_ = 34.0 min) was purified from subfraction B (16 mg) by utilizing semi-preparative, reverse-phase HPLC (YMC-Triart C18, 250 × 10.0 mm, 5 μm, at a flow rate of 2 mL/min) with an isocratic solvent system of 34% MeOH. Subfraction C (22 mg) was isolated by semi-preparative, reverse-phase HPLC (YMC-Triart C18, 250 × 10.0 mm, 5 μm, at a flow rate of 2 mL/min) with an isocratic solvent system of 38% MeOH to give compounds **6** (1.5 mg, *t*_R_ = 78.0 min) and **8** (2.5 mg, *t*_R_ = 51.0 min). Compounds **1** (1.5 mg, *t*_R_ = 42.0 min), **2** (2.5 mg, *t*_R_ = 26.5 min), **3** (28.0 mg, *t*_R_ = 48.0 min), **4** (1.2 mg, *t*_R_ = 25.0 min), and **7** (1.0 mg, *t*_R_ = 33.5 min) were obtained from subfraction D (72 mg) by using semi-preparative, reverse-phase HPLC (YMC-Triart C18, 250 × 10.0 mm, 5 μm, at a flow rate of 2 mL/min) with an isocratic solvent system of 60% MeOH.

#### 4.4.1. Brevicolactone A (**1**)

Amorphous powder; ${\left[\alpha \right]}_{D}^{25}$ + 27.1 (c 0.90, MeOH); UV (MeOH) *λ*_max_ (log ε) 214 (3.89), 250 (0.78), 304 (0.39); IR (KBr) *ν*_max_ 3725, 2951, 1688, 1275 cm^−1^; ^1^H (800 MHz); and ^13^C NMR (200 MHz), see Table 1; positive-HR-ESIMS *m/z* 315.1201 [M+Na]^+^ (calcd. for C_16_H_20_O_5_Na, 315.1203).

#### 4.4.2. Brevicolactone B (**2**)

Amorphous powder; ${\left[\alpha \right]}_{D}^{25}$ + 18.7 (c 0.60, MeOH); UV (MeOH) *λ*_max_ (log ε) 192 (2.83), 216 (3.96), 251 (1.37), 306 (0.84); IR (KBr) *ν*_max_ 3423, 2941, 1738, 1138 cm^−1^; ^1^H (800 MHz); and ^13^C NMR (200 MHz), see Table 1; positive-HR-ESIMS *m/z* 359.1107 [M+Na]^+^ (calcd. for C_17_H_20_O_7_Na, 359.1101).

### 4.5. Computational Analysis for ECD Simulation and Optical Rotation Values

A MacroModel module (version 2023-3, Schrödinger LLC) with the MMFF force field under mixed torsional/low-mode sampling conditions was used. The conformational search was conducted in the gas phase, applying an energy window cutoff of 20 kJ/mol and allowing up to 10,000 steps to ensure the sufficient exploration of the conformational space. The geometric minimization of the resulting structures was carried out using the Polak−Ribiere Conjugate Gradient (PRCG) method, with 10,000 iterations and a convergence threshold of 0.001 kJ (mol Å)^−1^, based on the root mean square (RMS) gradient. From this process, conformers within 20 kJ/mol of the lowest-energy structure were retained and further optimized using Tmolex 2023 (BIOVIA) with density functional theory (DFT) at the B3LYP/6-31G(d,p) level. Calculated optical rotations of different conformers were calculated using the DFT method at the B3LYP/6-31G(d,p) level. Subsequent ECD (Electronic Circular Dichroism) calculations for these conformers were performed using the same theoretical method and basis set. Simulated ECD spectra were obtained by superimposing individual electronic transitions, where Δ*E*_i_ and *R*_i_ represent the excitation energy and the rotatory strength of the *i* transition, respectively, and *σ* (set to 0.15 eV) denotes the bandwidth at 1/e of the peak height. Boltzmann-weighted ECD spectra, derived from the calculated Gibbs free energies of each conformer, are provided in the Supplementary Information, and the visualization of the final spectra was carried out using GraphPad Prism 8.0 (GraphPad Software, San Diego, CA, USA).

$$\Delta \varepsilon \left(E\right)=\frac{1}{2.297\times 10^{-39}}\frac{1}{\sqrt{2\pi \sigma }}\sum_{i}^{A}\Delta E_{i}R_{i}e^{{\left[-\left(E-\Delta E_{i}/2\sigma \right)\right]}^{2}}$$

### 4.6. The Computational Prediction of the Biological Effects

The potential interactions between specific proteins and all the isolated compounds (**1**–**8**) were assessed using STITCH (http://stitch.embl.de/, accessed on 2 August 2024). The organism was set to Homo sapiens, and when no exact compound match was available, structurally similar compounds with a Tanimoto score of 0.89 or higher were selected for the analysis. To determine the biological processes associated with the identified proteins, Enrichr (https://maayanlab.cloud/Enrichr/, accessed on 7 August 2024) was utilized to analyze pathway involvement based on the Elsevier Pathway Collection database.

### 4.7. Cell Culture

The HepG2 human hepatoma cell line (American Type Culture Collection, Manassas, VA, USA) was grown in Dulbecco’s Modified Eagle’s Medium (Welgene, Gyeongsan, Korea) containing 10% fetal bovine serum (Cytiva, Marlborough, MA, USA) and 1% penicillin/streptomycin (Welgene).

### 4.8. Cell Viability Measurement

Cell viability was measured using a Cell Counting Kit-8 (CCK-8) (Dojindo, Kumamoto, Japan), according to the manufacturer’s instructions. Briefly, the HepG2 cells were treated with compounds or a vehicle for 12 h. After the treatment period, the cells were further incubated with a CCK-8 reagent for 1 h, and the absorbance was measured at 450 nm using a microplate reader.

### 4.9. Immunoblot Analysis

The cells were lysed using an RIPA buffer supplemented with protease and phosphatase inhibitors (GenDepot, Baker, TX, USA), following the manufacturer’s guidelines. Equal amounts of protein were separated onto 6% or 8% polyacrylamide gels and subsequently transferred to nitrocellulose membranes (Cytiva, Marlborough, MA, USA). The detection of protein bands was performed using the Pierce ECL Western Blotting Substrate (Thermo Fisher Scientific). Primary antibodies against SREBP1 and β-Actin were obtained from Santa Cruz Biotechnology (Dallas, TX, USA). The primary antibody for ACC1, horseradish peroxidase (HRP)-linked anti-rabbit immunoglobulin G (IgG), and HRP-linked anti-mouse IgG were purchased from Cell Signaling Technology (Danvers, MA, USA).

### 4.10. Reverse Transcription and Quantitative Real-Time PCR (RT-qPCR)

Total RNA was extracted from the cultured cells using a RiboEx reagent (Geneall, Seoul, Korea) in accordance with the manufacturer’s protocol. Complementary DNA (cDNA) was synthesized from 1 ug of total RNA using the ReverTraAce cDNA synthesis kit (Toyobo, Osaka, Japan). Quantitative PCR was performed using SYBR green-based detection (Enzynomics, Daejeon, Korea) on a CFX Connect Real-Time PCR System (Bio-Rad, Hercules, CA, USA). Gene expression levels were determined using the 2^−ΔΔCt^ method, with *36B4* serving as the internal control for normalization. The primer sequences used were as follows: *36B4* forward, 5′-CGACCTGGAAGTCCAACTAC-3′; *36B4* reverse, 5′-ATCTGCTGCATCTGCTTG-3′; *SREBP1c* forward, 5’-TCGCGGAGCCATGGATT-3’; *SREBP1c* reverse, 5’-GGAAGTCACTGTCTTGGTTGTTGA-3’; and *FASN* forward, 5’-TCGTGGGCTACAGCATGG T-3’; *FASN* reverse, 5’-GCCCTCTGAAGTCGAAGAAGAA-3’; *ACC1* forward, 5’-TTCAGAGGCAGGGTGGGTTA-3’; *ACC1* reverse, 5’-ACATACTCGTTTGTGTCATAATTTGGT-3’.

### 4.11. Oil Red O Staining

Primary mouse hepatocytes were isolated from C57BL/6J mice using a two-step collagenase perfusion technique, as previously described [59]. The hepatocytes were treated with GW3965 (2.5 μM) for 24 h, following a 30 min pretreatment with the compounds. The cells were fixed for 15 min with 4% paraformaldehyde, followed by washing thrice with PBS. After 45 min of staining with 0.6% Oil Red O solution in 60% isopropyl alcohol, the cells were washed with 60% isopropyl alcohol and distilled water (three times each) and counterstained with hematoxylin. Positive areas in five randomly selected high-power fields were analyzed.

### 4.12. Statistical Analysis

All the results are presented as means ± SEM. The statistical analyses were conducted using GraphPad Prism version 7.0a (GraphPad Software, La Jolla, CA, USA). Comparisons between two groups were made using an unpaired Student’s t-test. For analyses involving more than two groups, one-way ANOVA was applied, followed by a Tukey’s multiple comparison test. A *p*-value of less than 0.05 was considered to be indicative of statistical significance.

## 5. Conclusions

We successfully identified eight secondary metabolites from a Hydractinia-associated fungus, P. brevicompactum MSW10-1, including brevicolactones A (**1**) and B (**2**). Through a combination of advanced spectroscopic techniques, such as NMR and HR-ESIMS analyses, we elucidated the chemical structures of brevicolactones A and B and determined their absolute configurations using quantum chemical ECD calculations. In our biological evaluation, the isolated compounds, particularly 3 and 7, significantly inhibited the expression of key lipogenic genes, including SREBP1c, FASN, and ACC1, in HepG2 cells. In addition, the compounds were shown to suppress lipid accumulation in primary mouse hepatocytes, as evidenced by Oil Red O staining. Our findings suggested evidence that isolated compounds would be promising candidates for the development of novel treatments for MASLD and related metabolic disorders. Future studies should focus on elucidating the precise molecular mechanisms by which these compounds inhibit lipogenic gene expression and lipid accumulation. Overall, our study contributes to the growing body of evidence supporting the use of marine-derived natural products in drug discovery, particularly for the treatment of metabolic diseases.

## Figures and Tables

**Table 1:** ^1^H NMR (800 MHz) and ^13^C NMR (200 MHz) data of **1** and **2** in CD_3_OD ^a,b^.

Position	1		2	
	*δ*_C_	*δ*_H_ (*J* in Hz)	*δ*_C_	*δ*_H_ (*J* in Hz)
1	173.5 s		169.5 s	
3	70.5 t	5.26 s	103.0 d	6.37 s
3a	146.7 s		144.3 s	
4	117.7 s		118.8 s	
5	164.7 s		163.7 s	
6	123.7 s		123.9 s	
7	154.6 s		152.9 s	
7a	107.6 s		107.2 s	
1′	21.9 t	2.64 m	22.2 t	3.42 d (7.0)
2′	33.1 t	1.59 m; 1.92 m	122.7 d	5.26 t (7.0)
3′	47.7 d	2.60 m	133.7 s	
4′	16.4 q	1.13 d (7.0)	14.7 q	1.83 s
5′	215.3 s		34.2 t	2.28 m
6′	27.9 q	2.17 s	32.3 t	2.37 m
7′			175.9 s	
4-CH_3_	11.1 q	2.16 s	9.54 q	2.27 s
5-OCH_3_	61.3 q	3.78 s	60.0 q	3.78 s

^a^ Coupling constants (in parentheses) are shown in Hz. ^b 13^C NMR data were assigned based on HSQC and HMBC experiments.

## Data Availability

The data are contained within the article and Supplementary Information.

## Supplementary Materials

The following are available online at https://www.mdpi.com/article/10.3390/md23070275/s1. Figure S1: The HR-ESIMS data of **1**. Figure S2: The ^1^H NMR spectrum of **1** (CD_3_OD, 800 MHz). Figure S3: The ^1^H-^1^H COSY spectrum of **1**. Figure S4: The HSQC spectrum of **1**. Figure S5: The HMBC spectrum of **1**. Figure S6: The HR-ESIMS data of **2**. Figure S7: The ^1^H NMR spectrum of **2** (CD_3_OD, 800 MHz). Figure S8: The ^1^H-^1^H COSY spectrum of **2**. Figure S9: The HSQC spectrum of **2**. Figure S10: The HMBC spectrum of **2**. Figure S11: The ROESY spectrum of **2**. Figure S12: The ^1^H NMR spectrum of **3** (CD_3_OD, 800 MHz). Figure S13: The ^1^H NMR spectrum of **4** (CD_3_OD, 800 MHz). Figure S14: The ^1^H NMR spectrum of **5** (CD_3_OD, 800 MHz). Figure S15: The ^1^H NMR spectrum of **6** (CD_3_OD, 800 MHz). Figure S16: The ^1^H NMR spectrum of **7** (CD_3_OD, 800 MHz). Figure S17: The ^1^H NMR spectrum of **8** (CD_3_OD, 800 MHz). Figure S18: Protein target prediction based on the chemical structures of the isolated compounds. Figure S19: The identification of biological pathways associated with the predicted target proteins using the Elsevier Pathway Collection. Figure S20: The inhibitory effects of compounds **3** and **7** on GW3965-induced *SREBP1c* expression in HepG2 cells. Figure S21: The evaluation of purity for compounds **1** and **2** by LC/MS (detection wavelength was set as 254 nm) Table S1: Calculated energy and Boltzmann distribution of compound **1a** conformer. Table S2: Calculated energy and Boltzmann distribution of compound **2a** conformers.

## References

[1] Rao G., Peng X., Li X., An K., He H., Fu X., Li S., An Z. (2023). Unmasking the enigma of lipid metabolism in metabolic dysfunction-associated steatotic liver disease: From mechanism to the clinic. Front. Med..

[2] Prasoppokakorn T. (2025). Applicability of statins in metabolic dysfunction-associated steatotic liver disease (MASLD). Livers.

[3] Reid M.V., Fredickson G., Mashek D.G. (2024). Mechanisms coupling lipid droplets to MASLD pathophysiology. Hepatology.

[4] Chandrasekaran P., Weiskirchen R. (2024). The role of SCAP/SREBP as central regulators of lipid metabolism in hepatic steatosis. Int. J. Mol. Sci..

[5] Li Y., Yang P., Ye J., Xu Q., Wu J., Wang Y. (2024). Updated mechanisms of MASLD pathogenesis. Lipids Health Dis..

[6] Rajewski P., Cieściński J., Rajewski P., Suwała S., Rajewska A., Potasz M. (2025). Dietary interventions and physical activity as crucial factors in the prevention and treatment of metabolic dysfunction-associated steatotic liver disease. Biomedicines.

[7] Latif S., Ahsan T. (2024). Prevalence of metabolic dysfunction-associated steatotic liver disease (MASLD) in persons with obesity and type 2 diabetes mellitus: A cross-sectional study. Euroasian J. Hepatogastroenterol..

[8] Smith K., Dennis K.M.J.H., Hodson L. (2024). The ins and outs of liver fat metabolism: The effect of phenotype and diet on risk of intrahepatic triglyceride accumulation. Exp. Physiol..

[9] Uehara K., Santoleri D., Whitlock A.E.G., Titchenell P.M. (2023). Insulin regulation of hepatic lipid homeostasis. Compr. Physiol..

[10] Wang S., Yin J., Liu Z., Liu X., Tian G., Xin X., Qin Y., Feng X. (2024). Metabolic disorders, inter-organ crosstalk, and inflammation in the progression of metabolic dysfunction-associated steatotic liver disease. Life Sci..

[11] Lekakis V., Papatheodoridis G.V. (2024). Natural history of metabolic dysfunction-associated steatotic liver disease. Eur. J. Intern. Med..

[12] Xiaoping Z., Fajun Y. (2012). Regulation of SREBP-mediated gene expression. Acta Biophys Sin..

[13] Li N., Li X., Ding Y., Liu X., Diggle K., Kisseleva T., Brenner D.A. (2023). SREBP regulation of lipid metabolism in liver disease, and therapeutic strategies. Biomedicines.

[14] Chang Y., Jeong S.W., Jang J.Y. (2023). Recent updates on pharmacologic therapy in non-alcoholic fatty liver disease. Clin. Mol. Hepatol..

[15] Santhiravel S., Dave D., Shahidi F. (2024). Bioactives from marine resources as natural health products: A review. Pharmacol. Rev..

[16] Li X., Xu H., Li Y., Liao S., Liu Y. (2023). Exploring diverse bioactive secondary metabolites from marine microorganisms using co-culture strategy. Molecules.

[17] Gan B., Wang K., Zhang B., Jia C., Lin X., Zhao J., Ding S. (2024). Dynamic microbiome diversity shaping the adaptation of sponge holobionts in coastal waters. Microbiol. Spectr..

[18] Wuerz M., Lawson C.A., Ueland M., Oakley C.A., Grossman A.R., Weis V.M., Suggett D.J., Davy S.K. (2022). Symbiosis induces unique volatile profiles in the model cnidarian Aiptasia. J. Exp. Biol..

[19] Boufridi A., Brinkmann C.M., Risdian C., Wink J., Kurtböke D.İ. (2024). Sponge symbiotic actinomycetes as sources of novel bioactive compounds atlantic and pacific ocean examples. Actinomycetes in Marine and Extreme Environments.

[20] Maire J., Philip G.K., Livingston J., Judd L.M., Blackall L.L., van Oppen M.J.H. (2023). Functional potential and evolutionary response to long-term heat selection of bacterial associates of coral photosymbionts. mSystems.

[21] Barzkar N., Sukhikh S., Babich O. (2024). Study of marine microorganism metabolites: New resources for bioactive natural products. Front. Microbiol..

[22] Garrett O., Whalen K.E. (2023). A bacterial quorum sensing signal is a potent inhibitor of de novo pyrimidine biosynthesis in the globally abundant *Emiliania huxleyi*. Front. Microbiol..

[23] Mazumder S., Bhattacharya D., Nag M., Lahiri D. (2024). Bioactive compounds from marine algae and fungi in down-regulating quorum sensing. Blue Biotechnol..

[24] Tan L.T. (2023). Impact of marine chemical ecology research on the discovery and development of new pharmaceuticals. Mar. Drugs.

[25] Wani S.N., Zahoor I. (2025). Bioactive compounds from marine organisms and their potential applications. Handbook of Research in Marine Pharmaceutics.

[26] Lasalo M., Jauffrais T., Georgel P., Matsui M. (2024). Marine microorganism molecules as potential anti-inflammatory therapeutics. Mar. Drugs.

[27] Lv F., Zeng Y. (2024). Novel bioactive natural products from marine-derived *Penicillium* fungi: A review (2021–2023). Mar. Drugs.

[28] Tang Y.-Q., Liang X., Zou Q.-H., Cui H., Luo L.-X., Qi S.-H. (2024). HPPO-derived meroterpenoids from the marine-derived fungus *Penicillium* sp. SCSIO 41691. J. Nat. Prod..

[29] Shaaban R., Elnaggar M.S., Khalil N., Singab A.N.B. (2023). A comprehensive review on the medicinally valuable endosymbiotic fungi *Penicillium chrysogenum*. Arch. Microbiol..

[30] Anh D.H., Tu N.T., Hanh T.T.H., Cuong N.X., Vien L.T., Ngan N.T.T., Ha D.V., Quang T.H. (2024). Terpenes and polyketides from the marine-derived fungus *Penicillium* sp. OPR23-FS02 with cytotoxic and antimicrobial effects. Vietnam J. Chem..

[31] Le V.-T., Bertrand S., Brandolini-Bunlon M., Gentil E., Du Pont T.R., Rabesaotra V., Wielgosz-Collin G., Mossion A., Grovel O. (2023). Global metabolome changes induced by environmentally relevant conditions in a marine-sourced *Penicillium restrictum*. C. R. Chim..

[32] Le V.-T., Bertrand S., Robiou du Pont T., Fleury F., Caroff N., Bourgeade-Delmas S., Gentil E., Logé C., Genta-Jouve G., Grovel O. (2021). Untargeted metabolomics approach for the discovery of environment-related pyran-2-ones chemodiversity in a marine-sourced *Penicillium restrictum*. Mar. Drugs.

[33] El-Hawary S.S., Sayed A.M., Mohammed R., Hassan H.M., Zaki M.A., Rateb M.E., Mohammed T.A., Amin E., Abdelmohsen U.R. (2018). Epigenetic modifiers induce bioactive phenolic metabolites in the marine-derived fungus *Penicillium brevicompactum*. Mar. Drugs.

[34] Vinale F., Salvatore M.M., Nicoletti R., Staropoli A., Manganiello G., Venneri T., Borrelli F., DellaGreca M., Salvatore F., Andolfi A. (2020). Identification of the main metabolites of a marine-derived strain of *Penicillium brevicompactum* using LC and GC MS techniques. Metabolites.

[35] Ye Y., Du L., Zhang X., Newmister S.A., McCauley M., Alegre-Requena J.V., Zhang W., Mu S., Minami A., Fraley A.E. (2020). Fungal-derived brevianamide assembly by a stereoselective semipinacolase. Nat. Catal..

[36] Lee S.R., Gallant É., Seyedsayamdost M.R. (2024). Discovery of cryptic natural products using high-throughput elicitor screening on agar media. Biochemistry.

[37] Lee S.R., Dayras M., Fricke J., Guo H., Balluff S., Schalk F., Yu J.S., Jeong S.Y., Morgenstern B., Slippers B. (2024). Molecular networking and computational NMR analyses uncover six polyketide-terpene hybrids from termite-associated *Xylaria* isolates. Commun. Chem..

[38] Lee S.R., Seyedsayamdost M.R. (2022). Induction of diverse cryptic fungal metabolites by steroids and channel blockers. Angew. Chem. Int. Ed..

[39] Han E.J., Jeong M., Lee S.R., Sorensen E.J., Seyedsayamdost M.R. (2024). Hirocidins, Cytotoxic Metabolites from *Streptomyces hiroshimensis*, Induce Mitochondrion-Mediated Apoptosis. Angew. Chem. Int. Ed..

[40] Li Y., Lee S.R., Han E.J., Seyedsayamdost M.R. (2022). Momomycin, an antiproliferative cryptic metabolite from the oxytetracycline producer *Streptomyces rimosus*. Angew. Chem. Int. Ed..

[41] Habib E., León F., Bauer J.D., Hill R.A., Carvalho P., Cutler H.G., Cutler S.J. (2008). Mycophenolic derivatives from *Eupenicillium parvum*. J. Nat. Prod..

[42] Jones D.F., Moore R.H., Crawley G.C. (1970). Microbial modification of mycophenolic acid. J. Chem. Soc. C..

[43] Danheiser R.L., Gee S.K., Perez J.J. (1986). Total synthesis of mycophenolic acid. J. Am. Chem. Soc..

[44] Canonica L., Rindone B., Santaniello E., Scolastico C. (1972). A total synthesis of mycophenolic acid, some analogues and some biogenetic intermediates. Tetrahedron.

[45] Grove J.F. (1972). New metabolic products of Aspergillus flavus. Part II. Asperflavin, anhydroasperflavin, and 5, 7-dihydroxy-4-methylphthalide. J. Chem. Soc., Perkin Trans..

[46] Kametani T., Kanaya N., Ihara M. (1980). Asymmetric total synthesis of brevianamide E. J. Am. Chem. Soc..

[47] Ali H., Ries M.I., Lankhorst P.P., van der Hoeven R.A.M., Schouten O.L., Noga M., Hankemeier T., van Peij N.N.M.E., Bovenberg R.A.L., Vreeken R.J. (2014). A non-canonical NRPS is involved in the synthesis of fungisporin and related hydrophobic cyclic tetrapeptides in *Penicillium chrysogenum*. PLoS ONE.

[48] Saeed A., Dullaart R.P.F., Schreuder T.C.M.A., Blokzijl H., Faber K.N. (2017). Disturbed vitamin A metabolism in non-alcoholic fatty liver disease (NAFLD). Nutrients.

[49] Zhong G., Kirkwood J., Won K.-J., Tjota N., Jeong H., Isoherranen N. (2019). Characterization of vitamin A metabolome in human livers with and without nonalcoholic fatty liver disease. J. Pharmacol. Exp. Ther..

[50] Othman A., Liu M., Bode H., Boudyguina E., von Eckardstein A., Parks J.S., Hornemann T. (2023). Hepatocyte ABCA1 deficiency is associated with reduced HDL sphingolipids. Front. Physiol..

[51] Choi H.Y., Choi S., Iatan I., Ruel I., Genest J. (2023). Biomedical advances in ABCA1 transporter: From bench to bedside. Biomedicines.

[52] Repa J.J., Liang G., Ou J., Bashmakov Y., Lobaccaro J.M., Shimomura I., Shan B., Brown M.S., Goldstein J.L., Mangelsdorf D.J. (2000). Regulation of mouse sterol regulatory element-binding protein-1c gene (SREBP-1c) by oxysterol receptors, LXRalpha and LXRbeta. Genes Dev..

[53] Jakobsson T., Treuter E., Gustafsson J.-Å., Steffensen K.R. (2012). Liver X receptor biology and pharmacology: New pathways, challenges and opportunities. Trends Pharmacol. Sci..

[54] Horton J.D., Goldstein J.L., Brown M.S. (2002). SREBPs: Activators of the complete program of cholesterol and fatty acid synthesis in the liver. J. Clin. Investig..

[55] Abdel-Magid A.F. (2015). Patent highlight: Fatty acid synthase (FASN) inhibitors as potential treatment for cancer, obesity, and liver related disorders. ACS Med. Chem. Lett..

[56] Griffett K., Burris T.P. (2023). Development of LXR inverse agonists to treat MAFLD, NASH, and other metabolic diseases. Front. Med..

[57] Doerfler D.L., Nulton C.P., Bartman C.D., Gottlieb F.J., Campbell I.M. (1978). Spore germination, colony development, and secondary metabolism in *Penicillium brevicompactum*: A radiogas chromatographic and morphological study. Can. J. Microbiol..

[58] Andersen B. (1991). Consistent production of phenolic compounds by *Penicillium brevicompactum* for chemotaxonomic characterization. Antonie Van Leeuwenhoek.

[59] Nguyen L.H., Cho Y.E., Kim S., Kim Y., Kwak J., Suh J.S., Lee J., Son K., Kim M., Jang E.S. (2024). Discovery of *N*-aryl-*N*′-[4-(aryloxy)cyclohexyl]squaramide-based inhibitors of LXR/SREBP-1c signaling pathway ameliorating steatotic liver disease: Navigating the role of SIRT6 activation. J. Med. Chem..

