# Supplementary material for: Isolation and Characterization of Secondary Metabolites from Hydractinia-Associated Fungus, Penicillium brevicompactum MSW10-1, and Their Inhibitory Effects on Hepatic Lipogenesis
Source: Mar Drugs. 2025 Jun 30;23(7):275. doi: 10.3390/md23070275 (PMC12300214; doi:10.3390/md23070275)
Supplement: Supplementary file 1 [file marinedrugs-23-00275-s001.zip › marinedrugs-3701804-supplementary.pdf]

Supporting Information

Isolation and Characterization of Secondary Metabolites from *Hydractinia*-associated fungus, *Penicillium brevicompactum* MSW10-1, and Their Inhibitory Effects on Hepatic Lipogenesis

Hyeon-Jeong Hwang[1,†], Hyeokjin Lim[1,†], Jae Sik Yu[2], Eun Seo Jang [1], Youngsang Nam [1], Yeo Jin Lee [1], Eun La Kim [1], Seonghwan Hwang[1,*], Seoung Rak Lee[1,*]

[1]*College of Pharmacy and Research Institute for Drug Development, Pusan National University, Busan 46241, Republic of Korea*

[2]*Department of Integrative Biological Sciences and Industry, Sejong University, Seoul 05006*

[†]These authors contributed equally to this study

*Corresponding authors

Supporting Information Contents:

Figure S1. The HR-ESIMS data of **1**……………………3
Figure S2. The ^{1}H NMR spectrum of **1** (CD_3OD, 800 MHz)……………………4
Figure S3. The ^{1}H-^{1}H COSY spectrum of **1**……………………5
Figure S4. The HSQC spectrum of **1**……………………6
Figure S5. The HMBC spectrum of **1**……………………7
Figure S6. The HR-ESIMS data of **2**……………………8
Figure S7. The ^{1}H NMR spectrum of **2** (CD_3OD, 800 MHz)……………………9
Figure S8. The ^{1}H-^{1}H COSY spectrum of **2**……………………10
Figure S9. The HSQC spectrum of **2**……………………11
Figure S10. The HMBC spectrum of **2**……………………12
Figure S11. The ROESY spectrum of **2**……………………13
Figure S12. The ^{1}H NMR spectrum of **3** (CD_3OD, 800 MHz)……………………14
Figure S13. The ^{1}H NMR spectrum of **4** (CD_3OD, 800 MHz)……………………15
Figure S14. The ^{1}H NMR spectrum of **5** (CD_3OD, 800 MHz)……………………16
Figure S15. The ^{1}H NMR spectrum of **6** (CD_3OD, 800 MHz)……………………17
Figure S16. The ^{1}H NMR spectrum of **7** (CD_3OD, 800 MHz)……………………18
Figure S17. The ^{1}H NMR spectrum of **8** (CD_3OD, 800 MHz)……………………19
Figure S18. Protein target prediction based on the chemical structures of the isolated compounds……………………20
Figure S19. The identification of biological pathways associated with the predicted target proteins using the Elsevier Pathway Collection……………………21
Figure S20. The inhibitory effects of compounds **3** and **7** on GW3965-induced *SREBP1c* expression in HepG2 cells……………………22
Figure S21. The evaluation of purity for compounds **1** and **2** by LC/MS (detection wavelength was set as 254 nm)……………………23
Table S1. Calculated energy and Boltzmann distribution of compound **1a** conformers……………………24
Table S2. Calculated energy and Boltzmann distribution of compound **2a** conformers……………………25

Figure S1. The HR-ESIMS data of **1**.

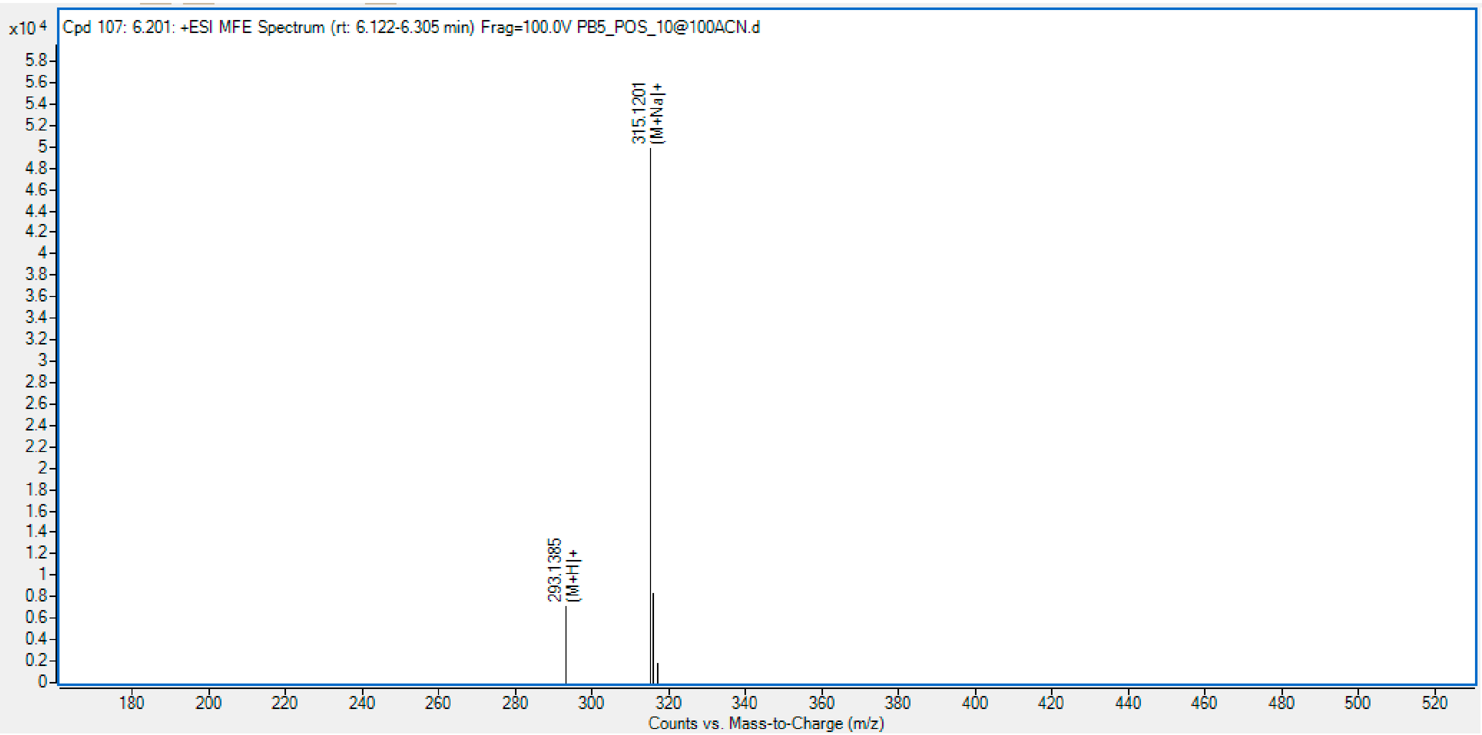

Figure S2. The ^{1}H NMR spectrum of **1** (CD_3OD, 800 MHz).

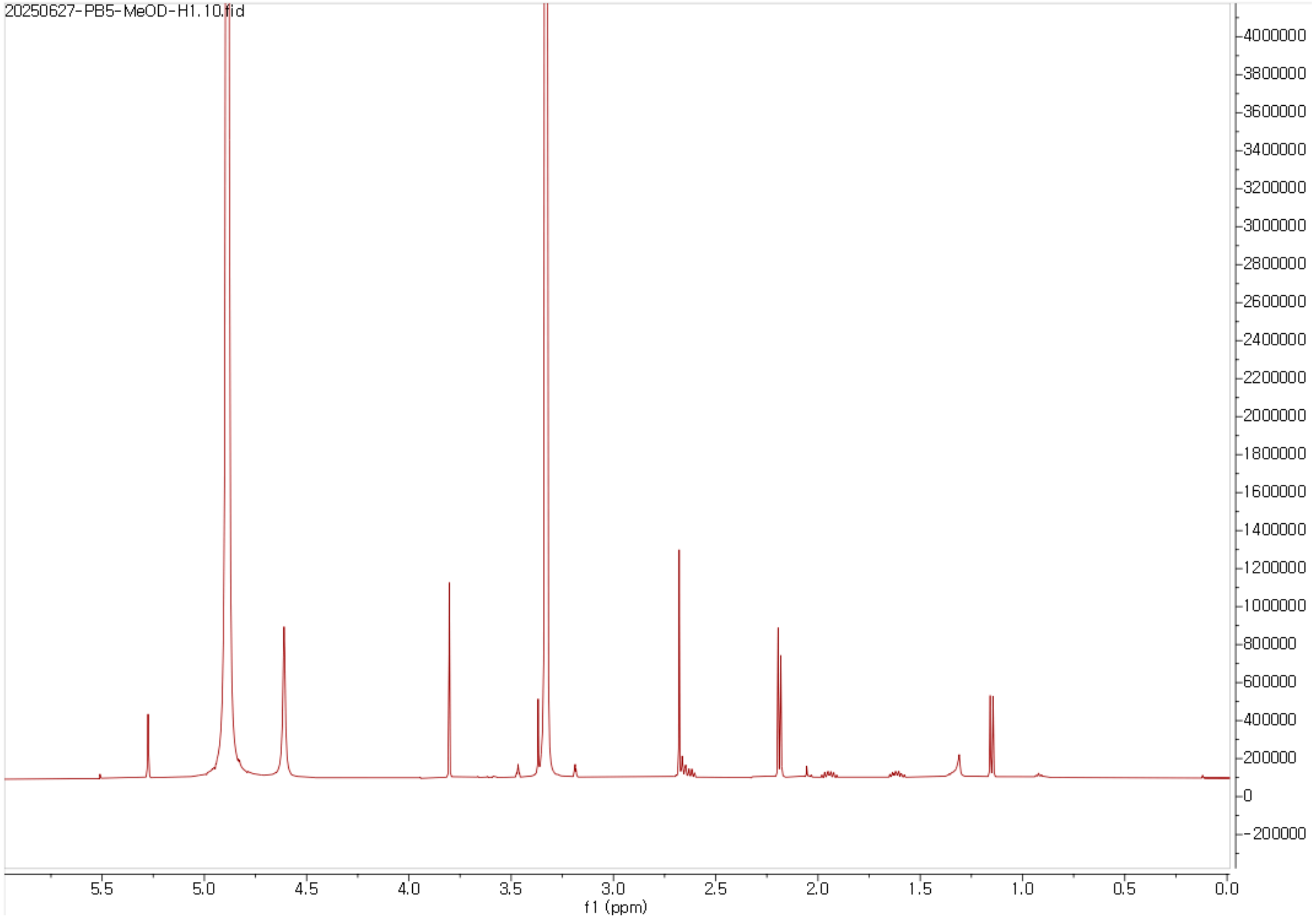

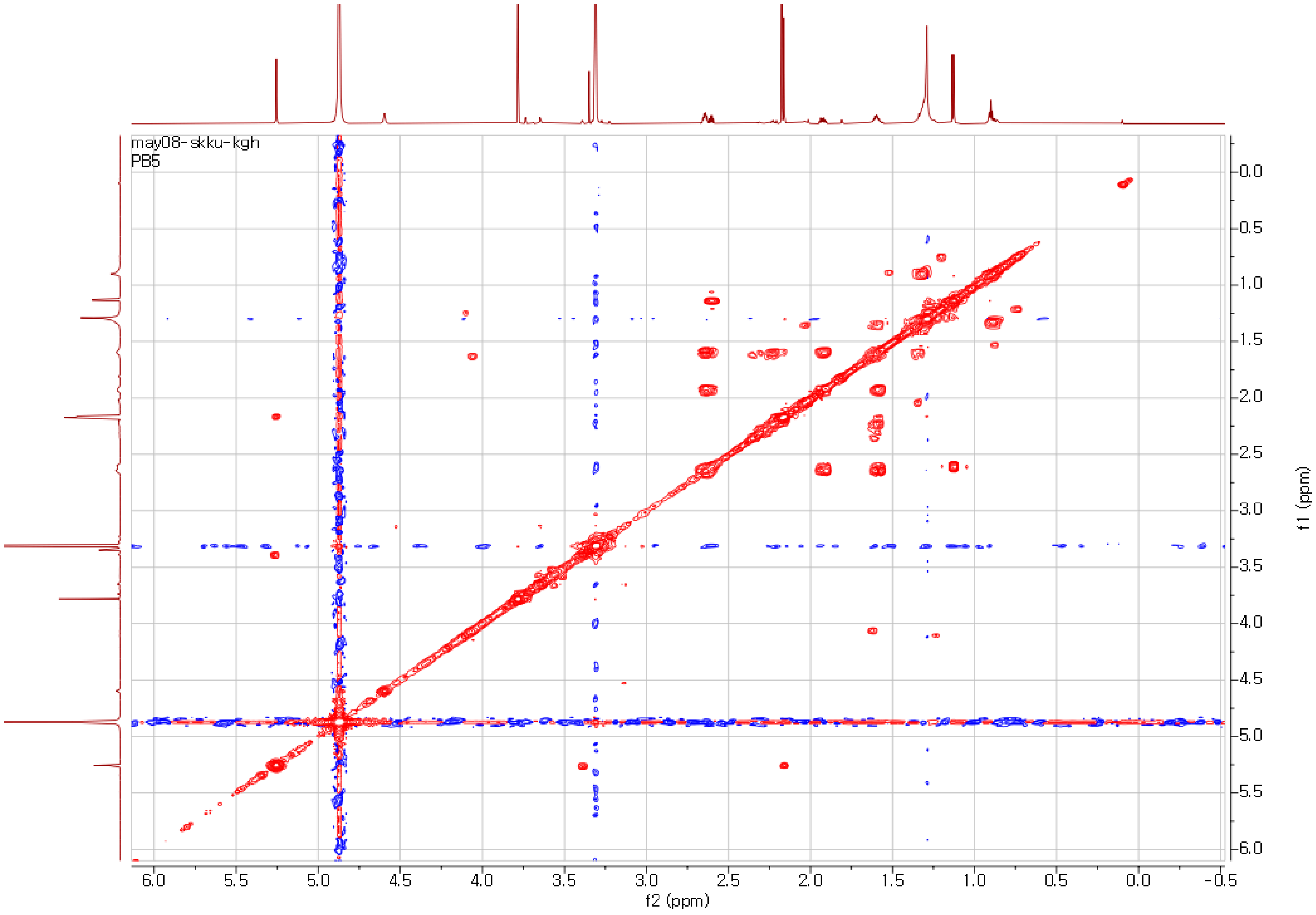

Figure S3. The ^{1}H-^{1}H COSY spectrum of **1.**

Figure S4. The HSQC spectrum of **1.**

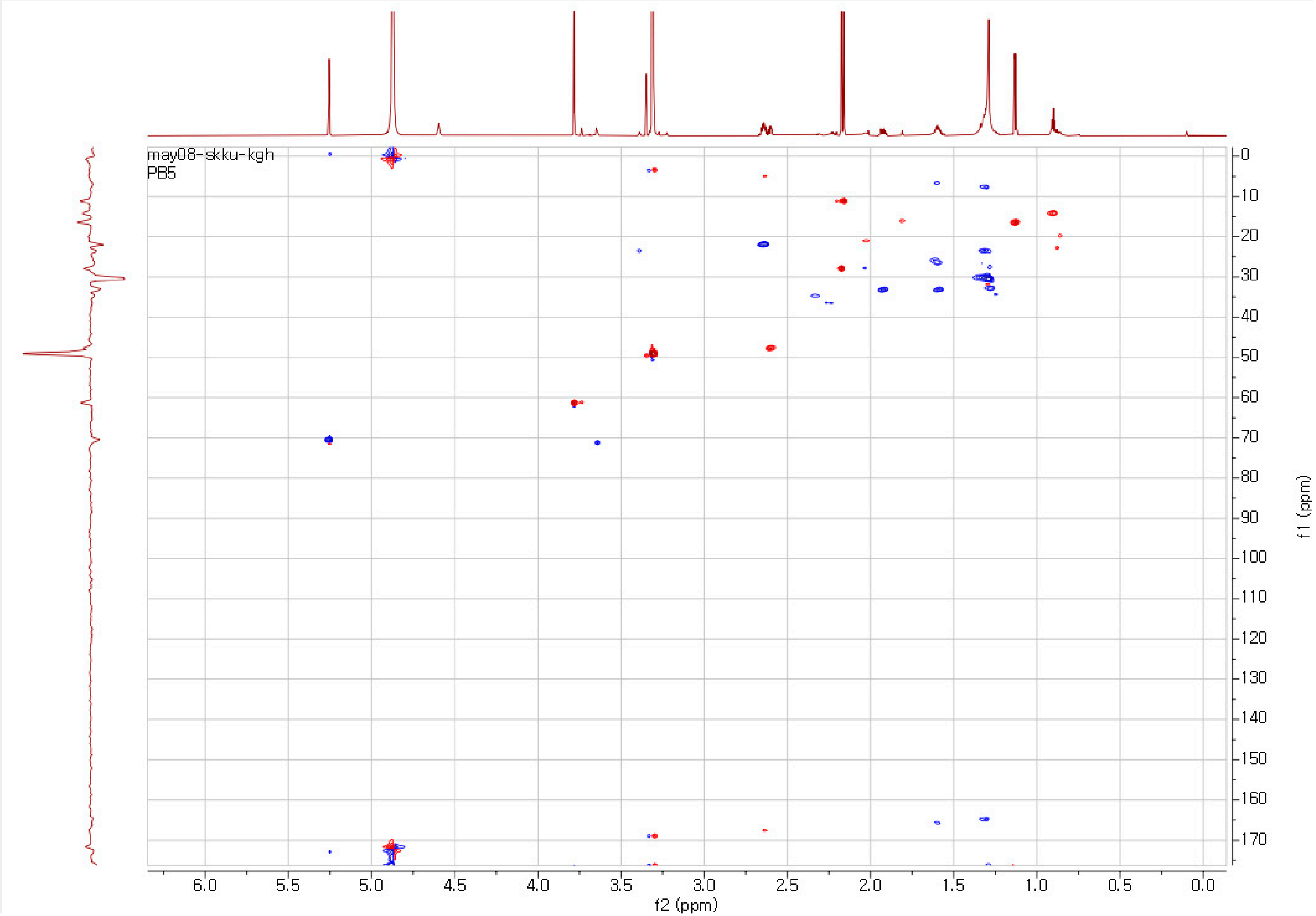

Figure S5. The HMBC spectrum of **1.**

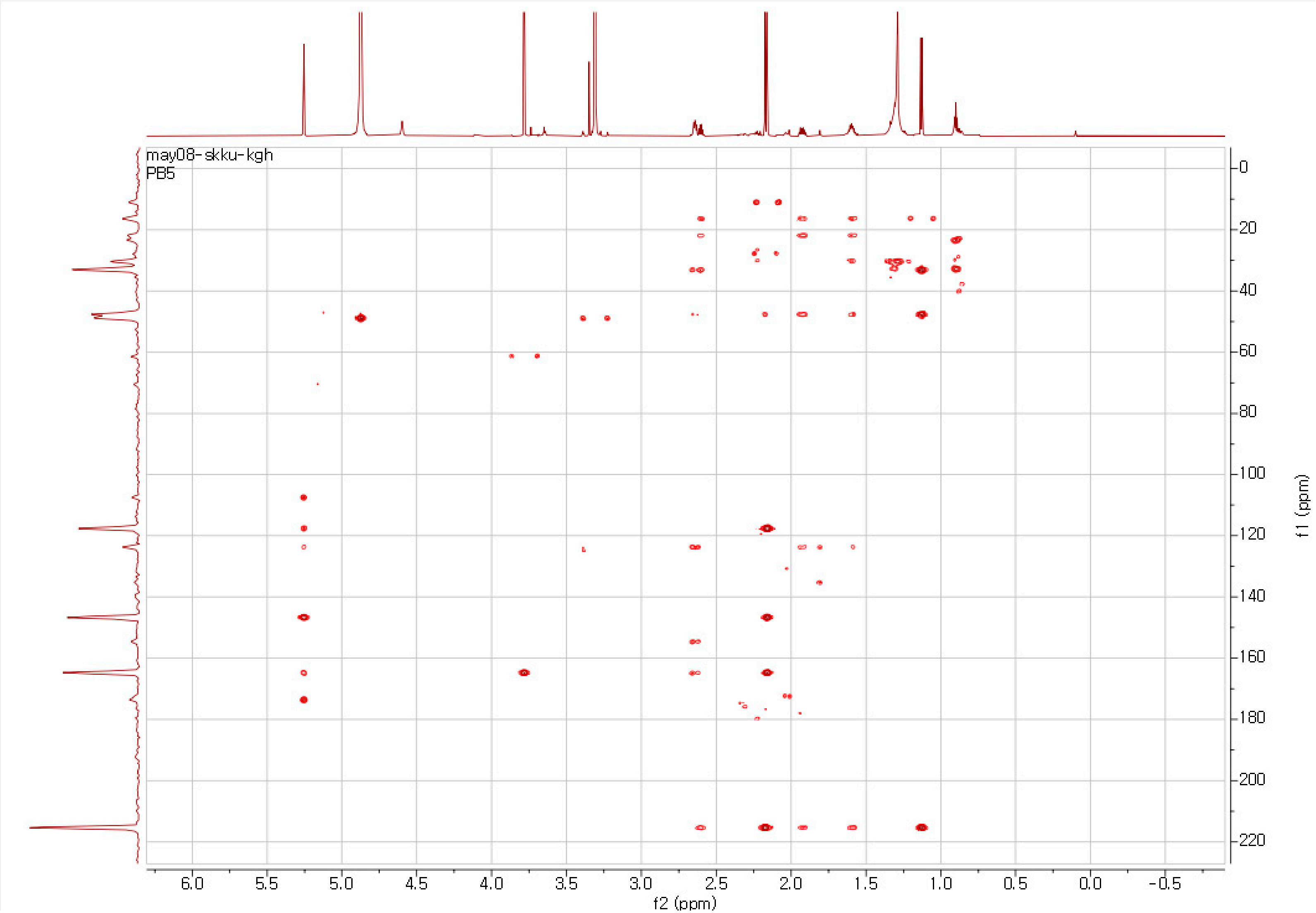

Figure S6. The HR-ESIMS data of **2.**

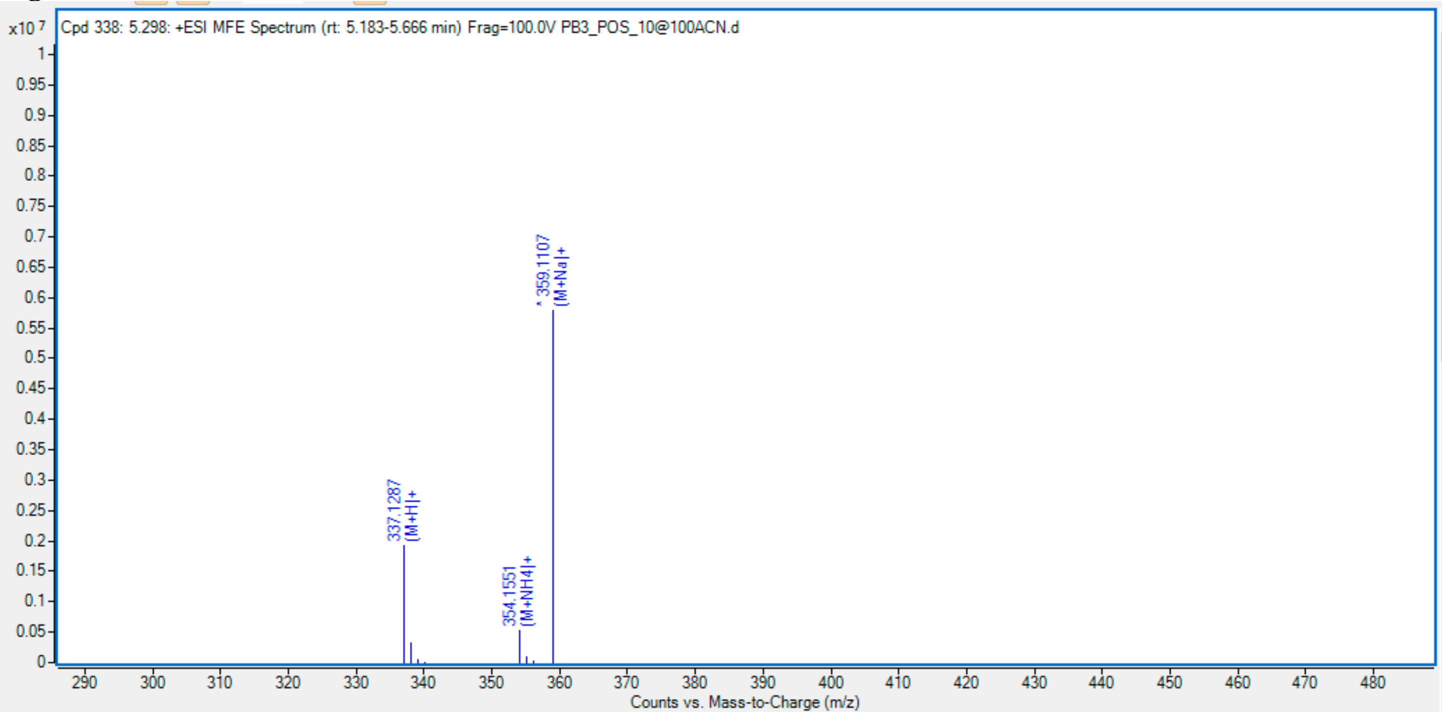

Figure S7. The ^{1}H NMR spectrum of **2** (CD_3OD, 800 MHz).

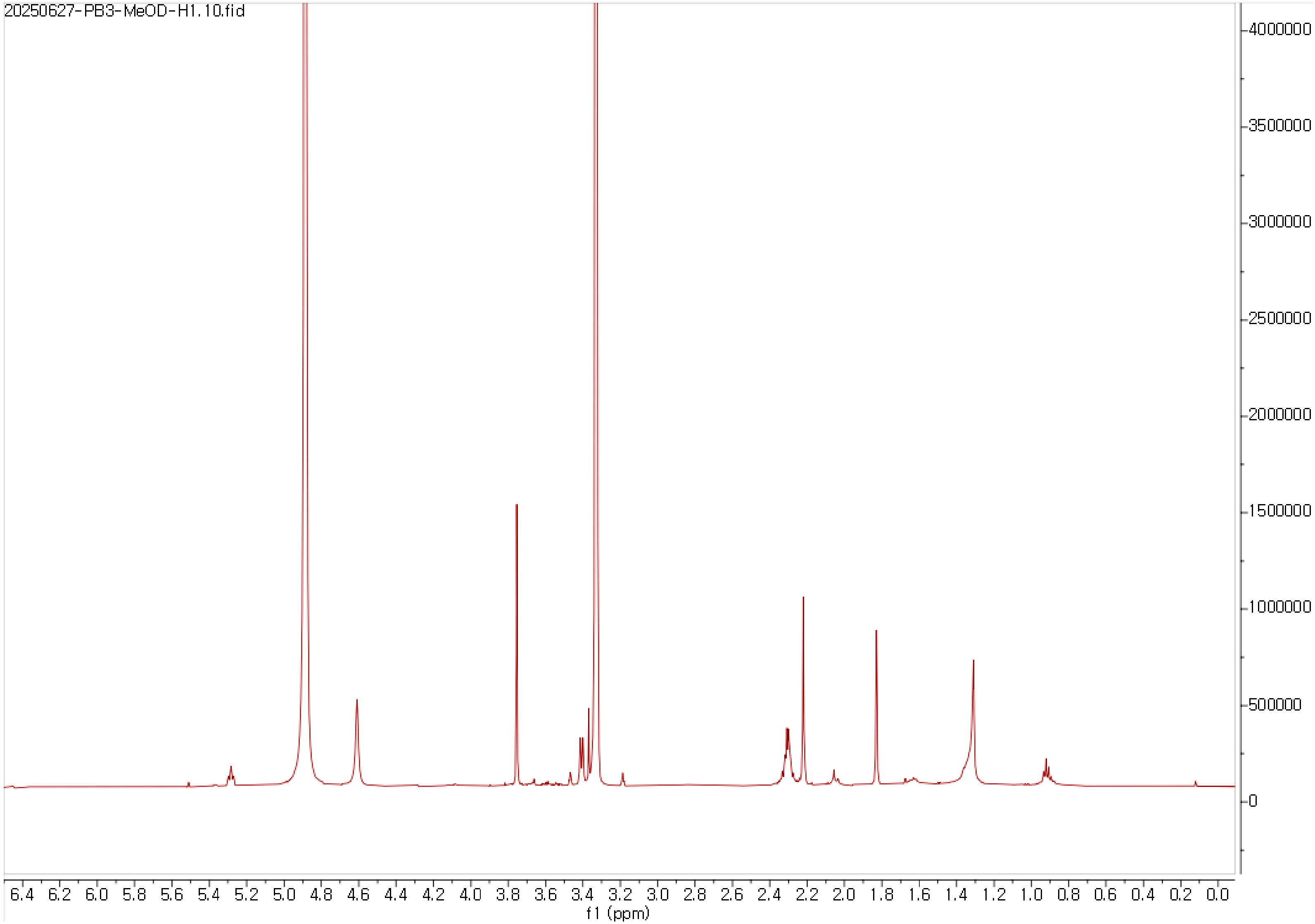

Figure S8. The ^{1}H-^{1}H COSY spectrum of **2.**

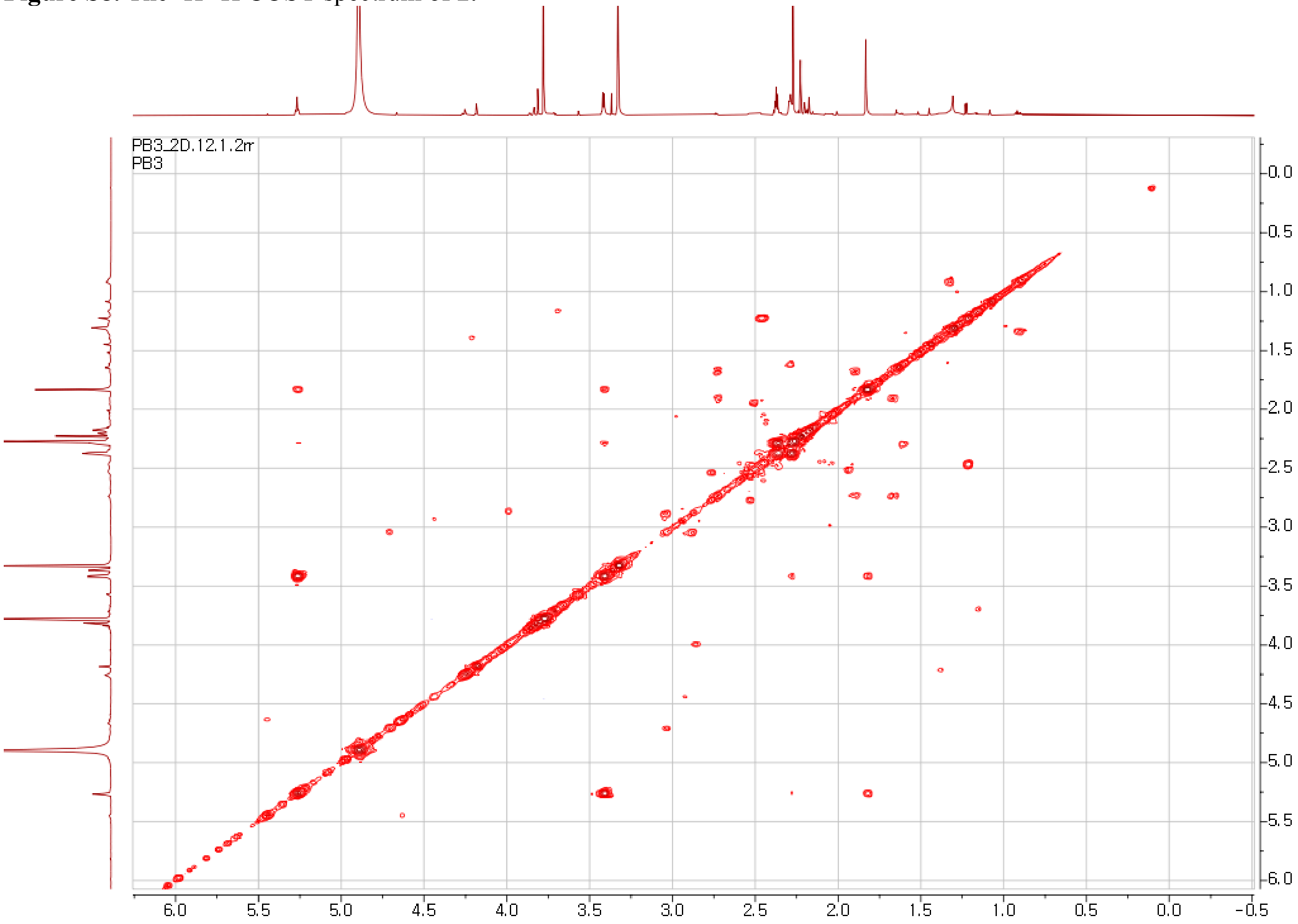

Figure S9. The HSQC spectrum of **2.**

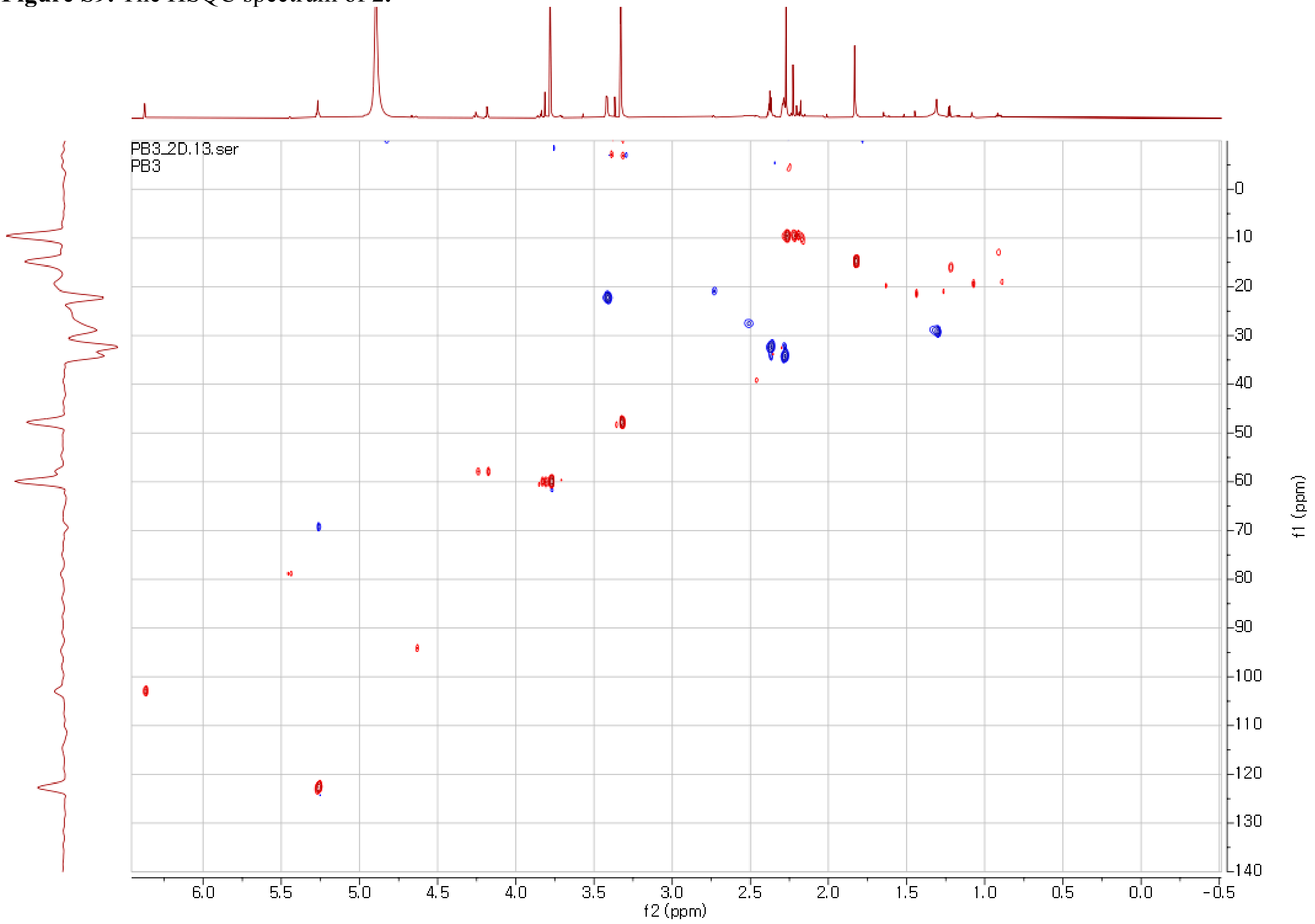

Figure S10. The HMBC spectrum of **2.**

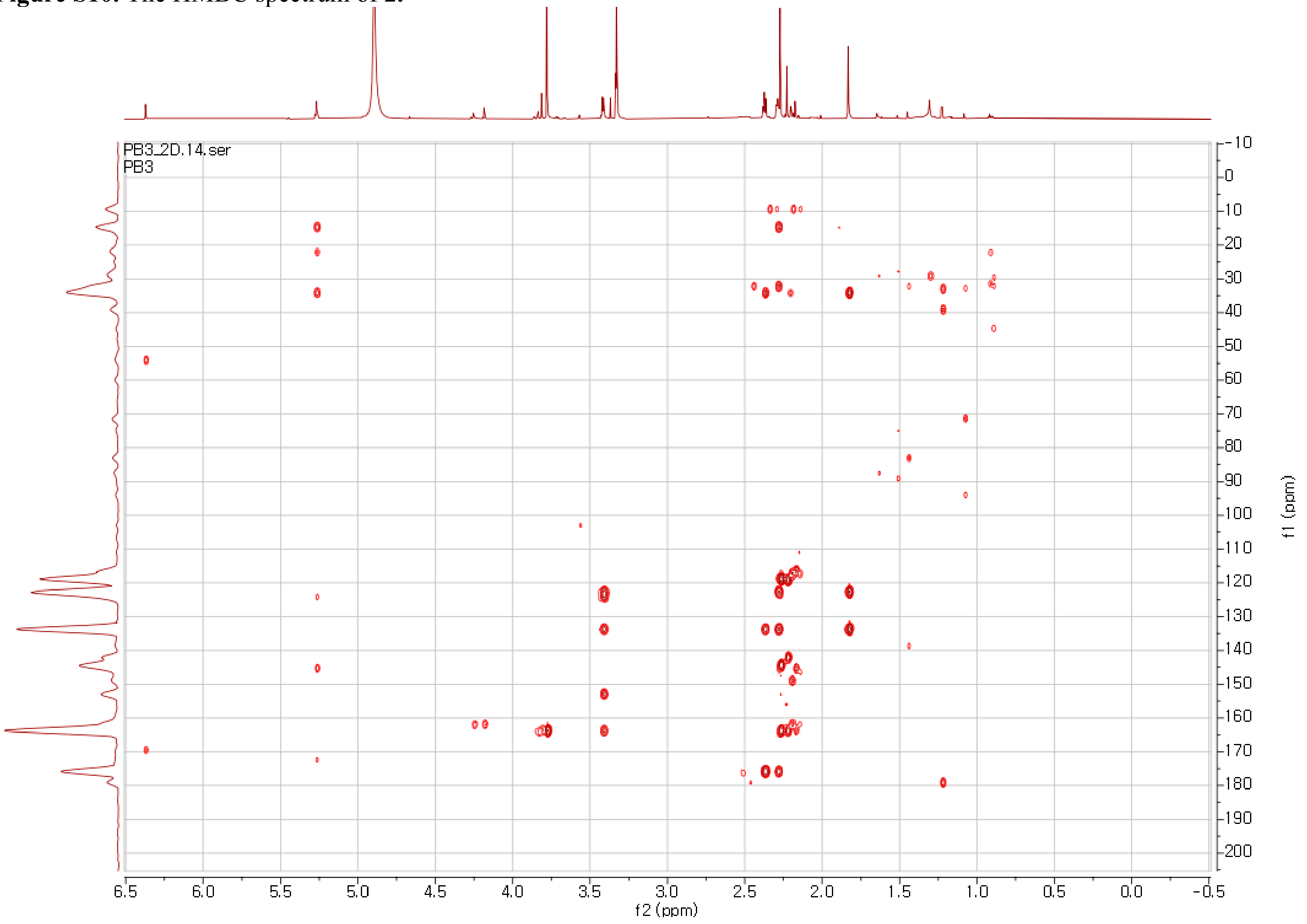

Figure S11. The ROESY spectrum of **2.**

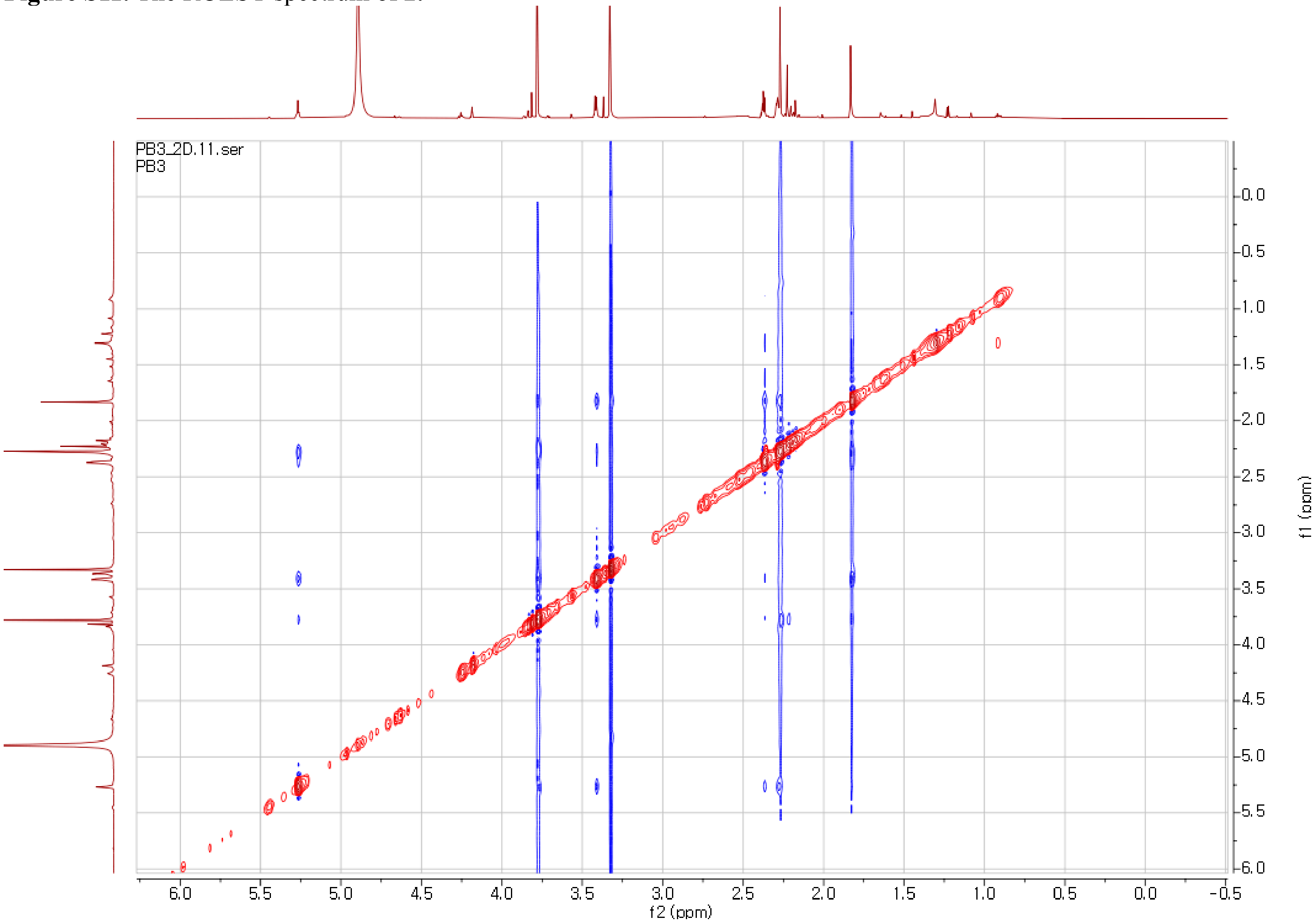

Figure S12. The ^{1}H NMR spectrum of **3** (CD_3OD, 800 MHz).

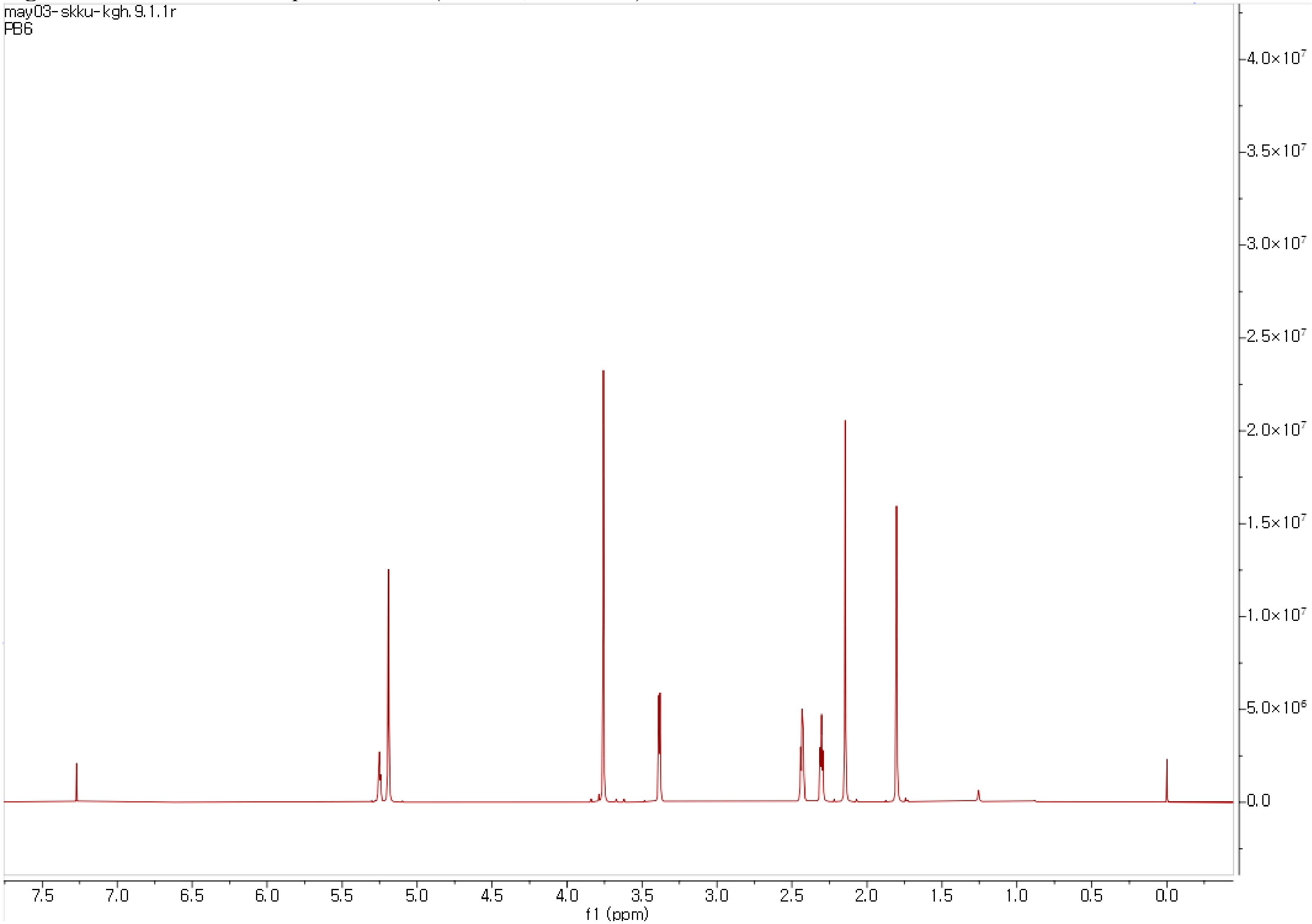

Figure S13. The ^{1}H NMR spectrum of **4** (CD_3OD, 800 MHz).

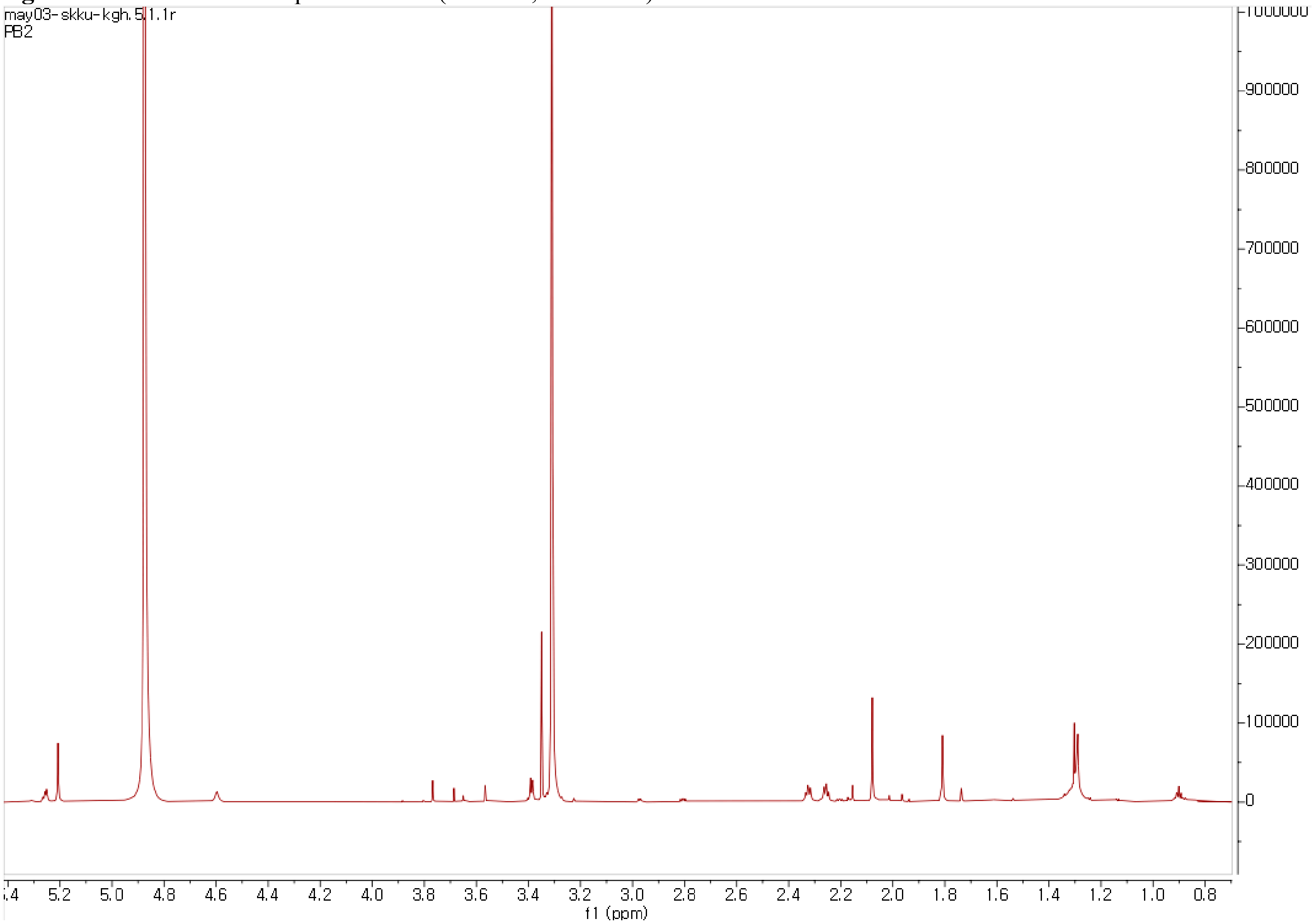

Figure S14. The ^{1}H NMR spectrum of **5** (CD_3OD, 800 MHz).

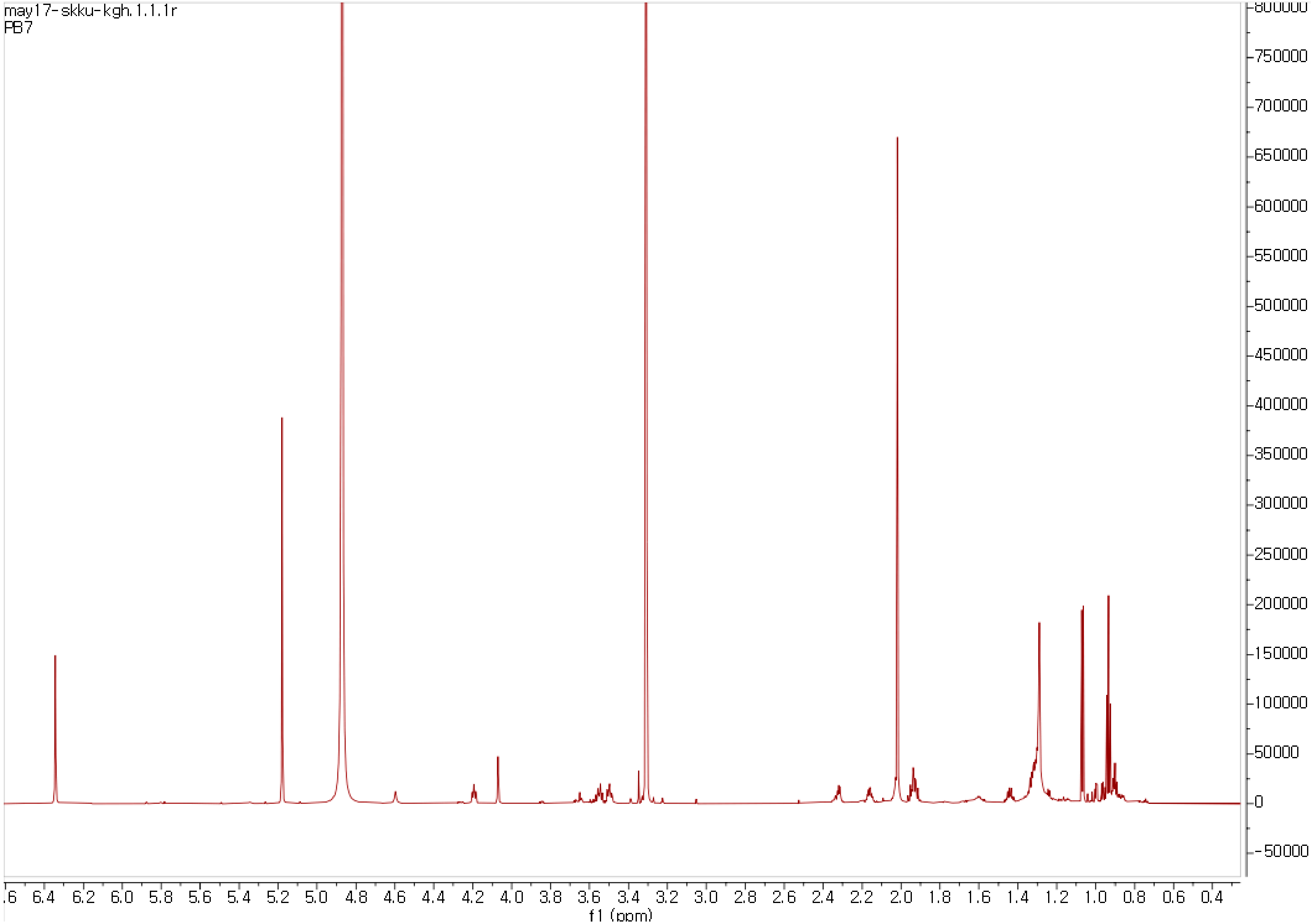

Figure S15. The ^1H NMR spectrum of **6** (CD_3OD, 800 MHz).

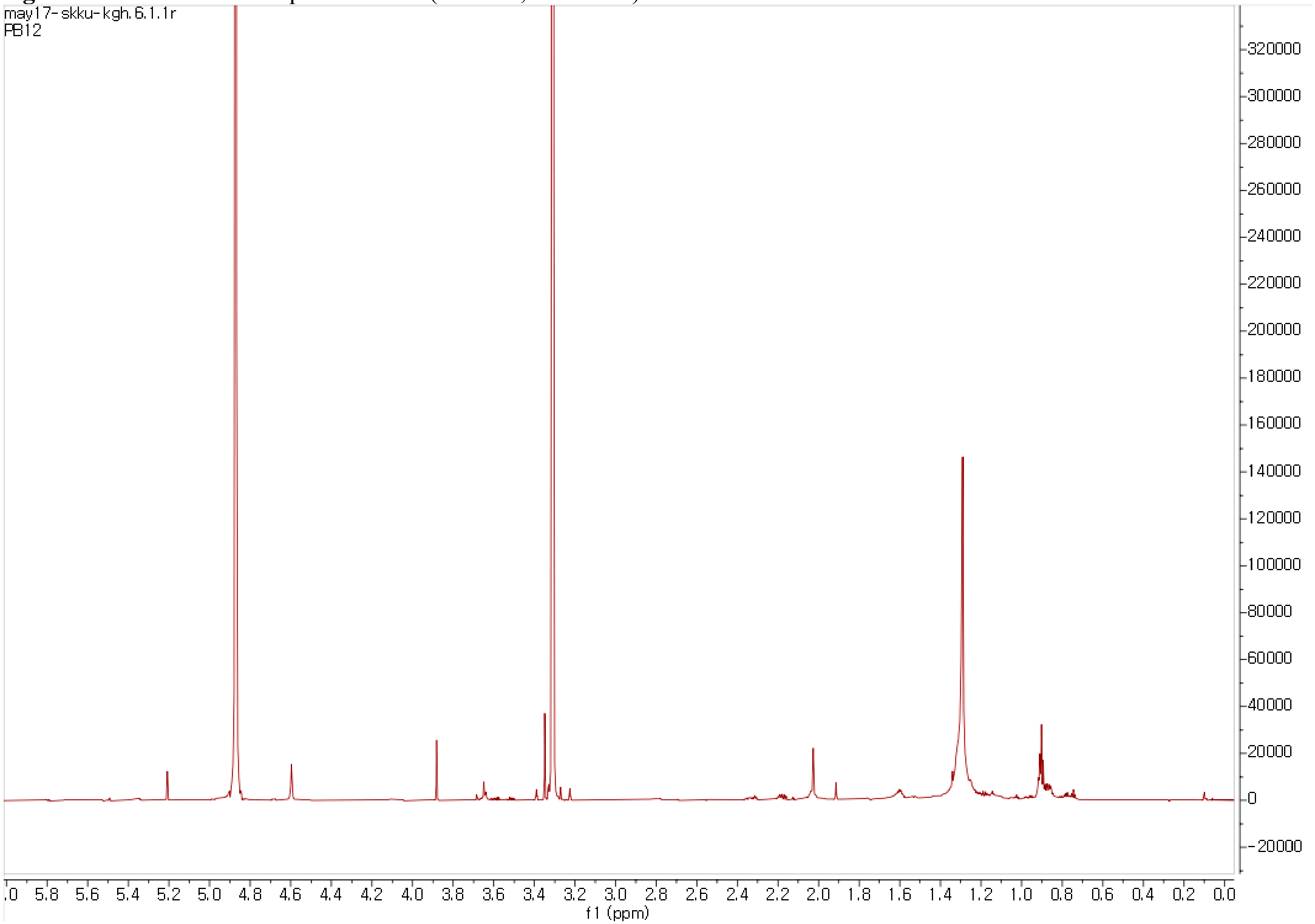

Figure S16. The ^{1}H NMR spectrum of **7** (CD_3OD, 800 MHz).

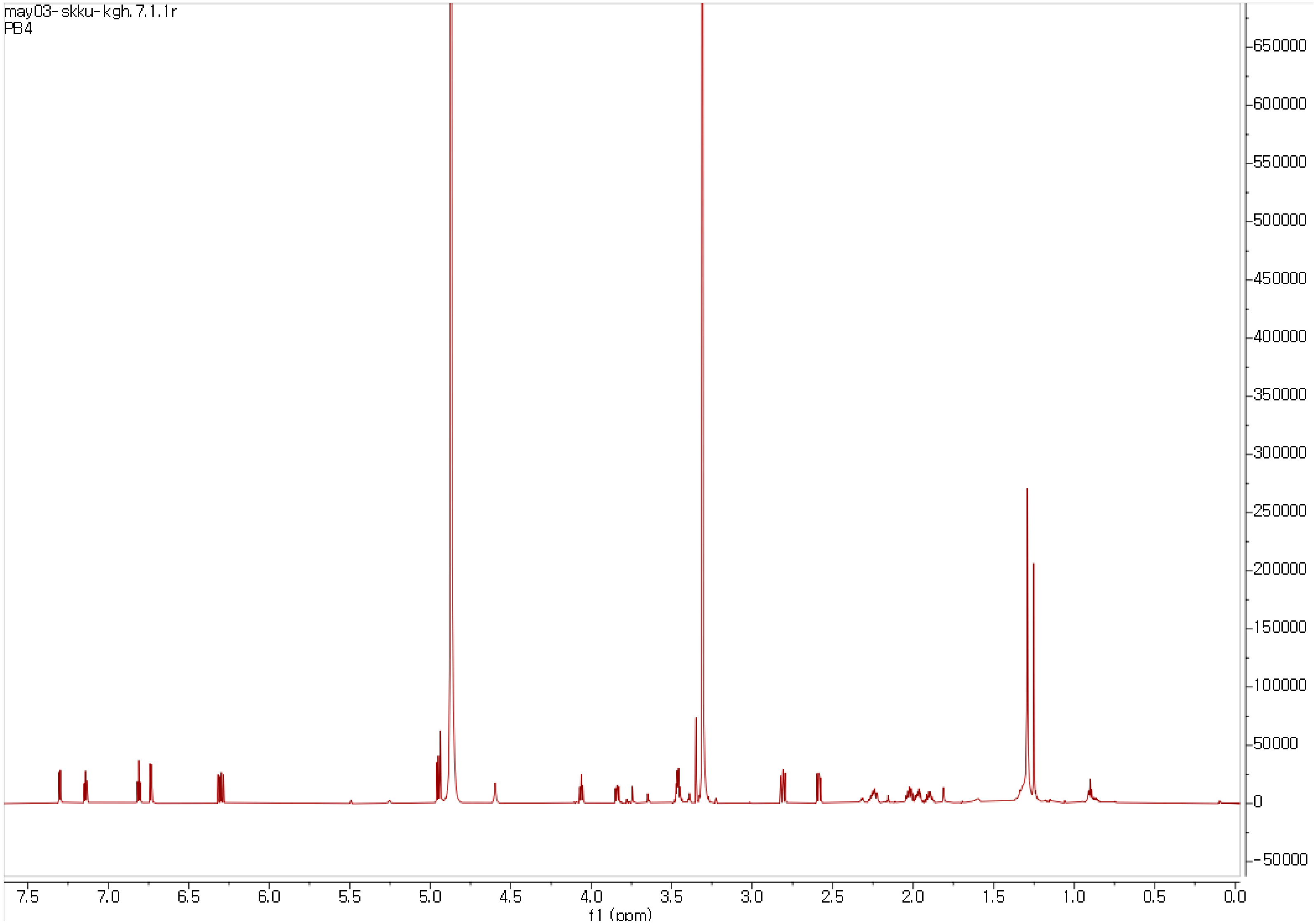

Figure S17. The ^{1}H NMR spectrum of **8** (CD_3OD, 800 MHz).

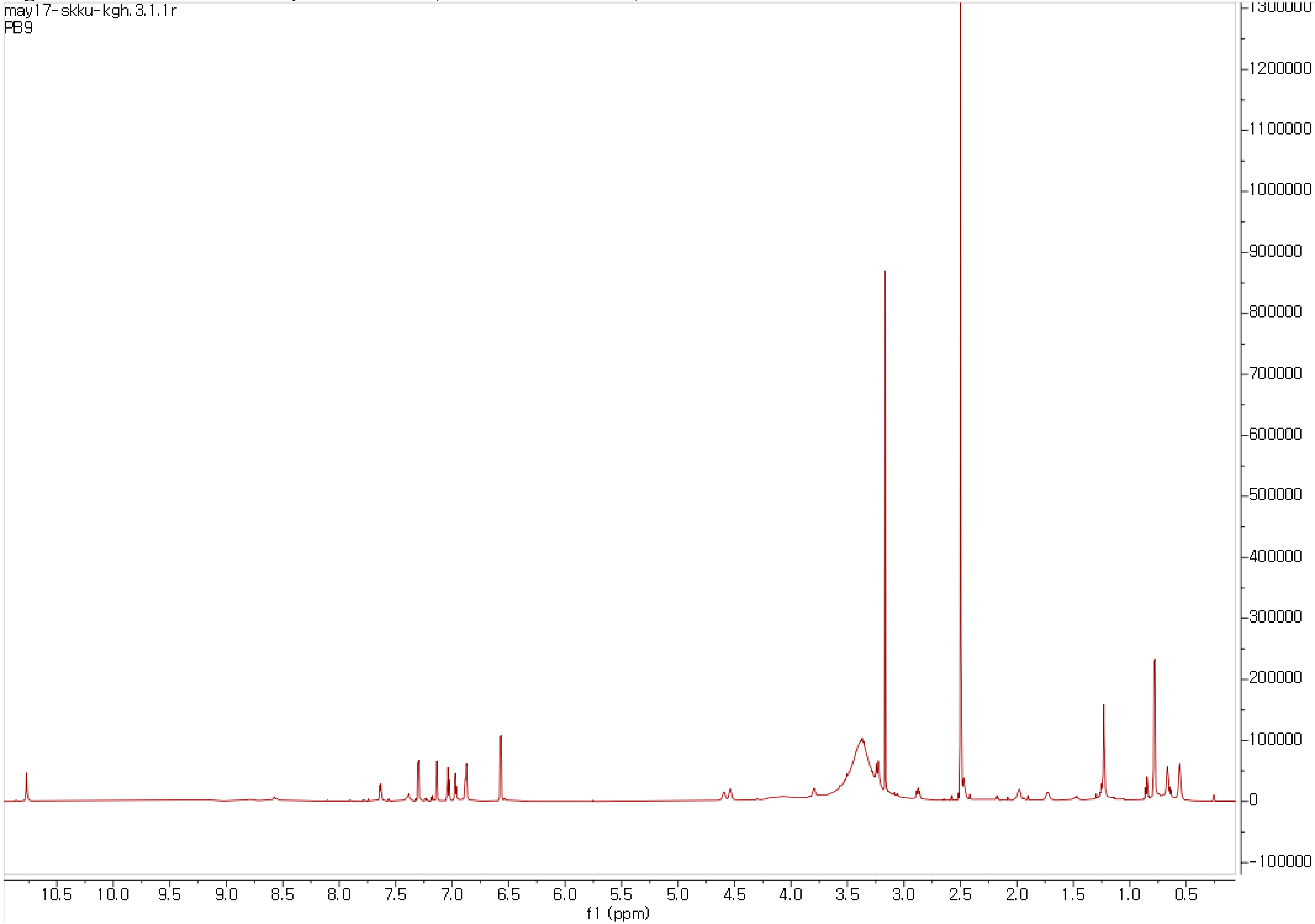

Figure S18. Protein target prediction based on the chemical structures of the isolated compounds.

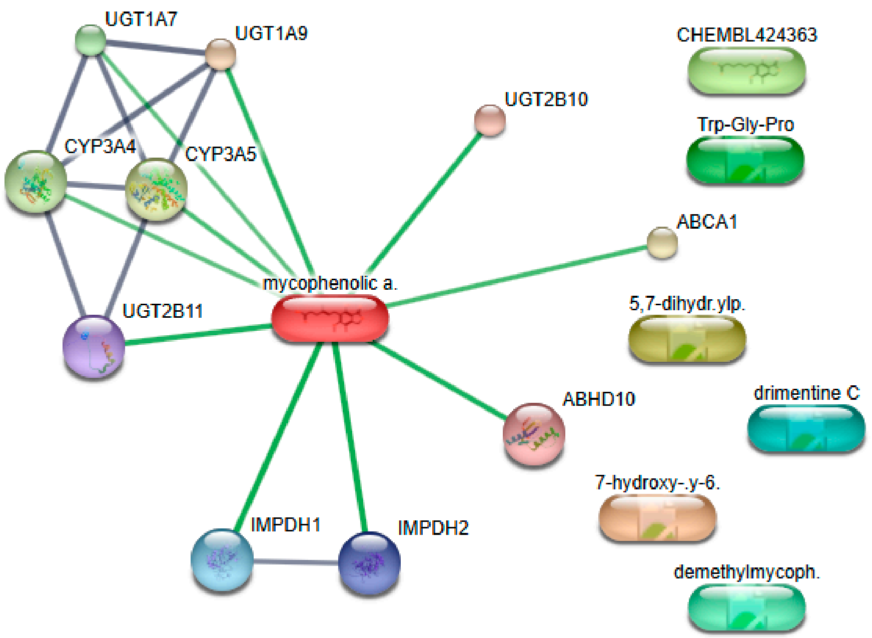

The compounds were analyzed using a computational prediction tool, and the resulting targets were visualized as a network. Green lines indicate predicted interactions between the compounds and target proteins, such as IMPDH1, CYP3A5, and UGT2B10, while gray lines represent known protein–protein interactions.

Figure S19. The identification of biological pathways associated with the predicted target proteins using the Elsevier Pathway Collection.

Elsevier Pathway Collection Bar Graph Table Clustergram Appyter

Hover each row to see the overlapping genes.

10 entries per page Search:

Index	Name	P-value	Adjusted p-value	Odds Ratio	Combined score
1	Vitamin A (Retinol) Metabolism and Visual Cycle	0.00009715	0.001166	178.23	1646.74
2	Proteins Involved in non-Alcoholic Fatty Liver Disease	0.003686	0.01597	26.91	150.79
3	Proteins Involved in Tangier Disease	0.003994	0.01597	317.19	1751.86
4	Find me Signal: Apoptotic Cell Attracts Phagocyte	0.008469	0.01991	138.71	661.82
5	Proteins Involved in Antiphospholipid Syndrome	0.008966	0.01991	130.54	615.43
6	Amyloid beta and APP Intracellular Transport in Alzheimer Disease	0.009957	0.01991	116.79	538.34
7	Lipids Enhance Apoptotic Cell Engulfment and Reduce Inflammation	0.01243	0.02131	92.44	405.55
8	Cortisol in Resolving Inflammation	0.02473	0.03709	45.22	167.30
9	Genes with Mutations Associated with Retinitis Pigmentosa	0.02814	0.03752	39.55	141.22
10	Proteins Involved in Retinitis Pigmentosa	0.06173	0.06889	17.52	48.78

Showing 1 to 10 of 12 entries | Export entries to table Previous Next

Terms marked with an * have an overlap of less than 5

Predicted protein targets were analyzed for potential physiological associations using a computational prediction tool. A functional pathway enrichment analysis was subsequently performed using the Elsevier Pathway Collection, revealing biologically relevant pathways associated with the predicted targets.

Figure S20. The inhibitory effects of compounds **3** and **7** on GW3965-induced *SREBP1c* expression in HepG2 cells.

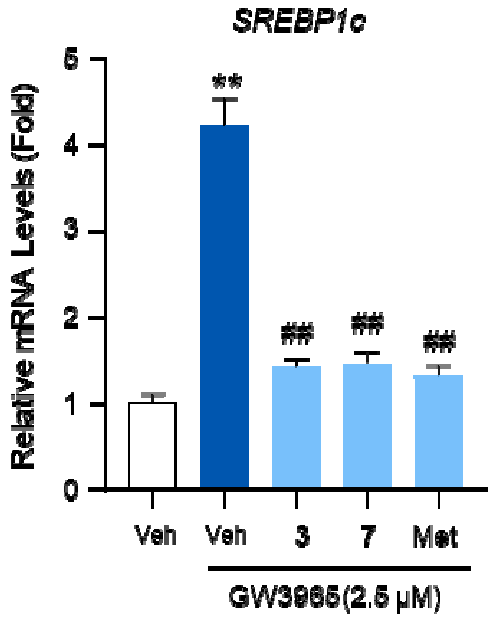

HepG2 cells were treated with GW3965 (2.5 μM) for 12 h, followed by a 30 min pretreatment with the compounds (10 μM) or metformin (10 mM). DMSO (0.1%) was used as vehicle. RNA extracted from the cells was subjected to RT-qPCR analysis of *SREBP1c*. In this, 36B4 was used as a reference gene. Values represent means ± SEM. A statistical evaluation was performed by one-way ANOVA with Tukey's post hoc test for multiple comparisons (**p<0.01 versus vehicle control; ##p<0.01 versus GW3965-treated condition). Veh, vehicle; Met, metformin.

Figure S21. The evaluation of purity for compounds **1** and **2** by LC/MS (detection wavelength was set as 254 nm).

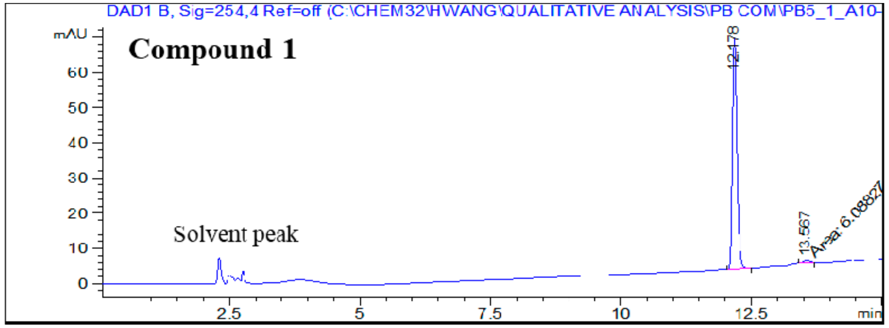

#	Time	Area	Height	Width	Area%	Symmetry
1	12.178	393.8	65.4	0.0923	98.478	0.743
2	13.567	6.1	6.3E-1	0.1619	1.522	1.036

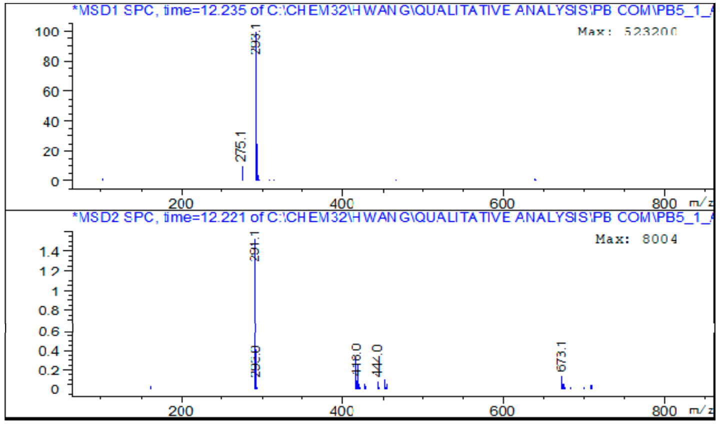

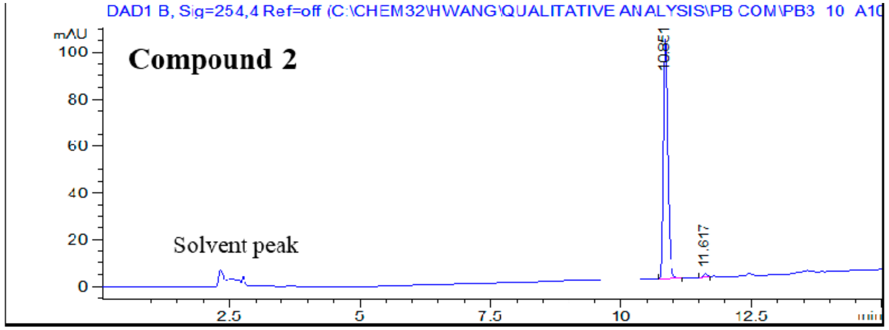

#	Time	Area	Height	Width	Area%	Symmetry
1	10.851	573	101.9	0.0876	98.697	0.745
2	11.617	7.6	1.4	0.0844	1.303	0.922

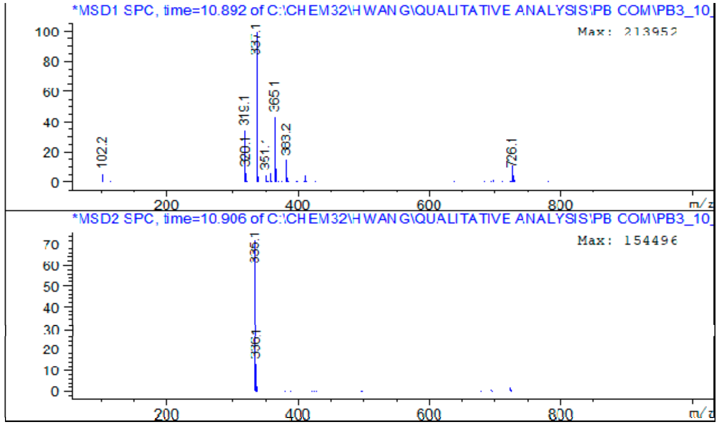

An analysis was performed by the injection of 1 µL of the sample by LC/MS analysis. The mobile phase consisting of formic acid in H_2O [0.1% (v/v)] (A) and acetonitrile [0.1% (v/v)] (B) was delivered at a flow rate of 0.3 mL/min by applying the following programmed gradient elution, 10-100% (B) for 15min, 100% (B) isocratic for 4 min, and then 10% (B) isocratic for 5 min, to perform the post-run reconditioning of the column (Agilent SB-C18, 50.0 × 2.1 mm, 5 µm).

Table S1. Calculated energy and Boltzmann distribution of compound **1a** conformers.

Conformers	B3-LYP/6-31G(d,p) Gibbs free energy (298.15 K) Calculated Energy (Hartree)	Relative Energy (kcal/mol)	Boltzmann population (%)
**1-1**	-1185.784177	0.00	22.83
**1-2**	-1185.783479	0.44	10.90
**1-3**	-1185.783434	0.47	10.39
**1-4**	-1185.783204	0.61	8.15
**1-5**	-1185.782952	0.77	6.24
**1-6**	-1185.782711	0.92	4.83
**1-7**	-1185.782684	0.94	4.70
**1-8**	-1185.782639	0.97	4.48
**1-9**	-1185.782632	0.97	4.45
**1-10**	-1185.782560	1.01	4.12
**1-11**	-1185.782394	1.12	3.45
**1-12**	-1185.782249	1.21	2.96
**1-13**	-1185.782237	1.22	2.92
**1-14**	-1185.782050	1.33	2.40
**1-15**	-1185.782016	1.36	2.31
**1-16**	-1185.781792	1.50	1.83
**1-17**	-1185.781280	1.82	1.06
**1-18**	-1185.781240	1.84	1.02
**1-19**	-1185.781189	1.88	0.96

Table S2. Calculated energy and Boltzmann distribution of compound **2a** conformers.

	B3-LYP/6-31G(d,p) Gibbs free energy (298.15 K)		
Conformers	Calculated Energy (Hartree)	Relative Energy (kcal/mol)	Boltzmann population (%)
**2-1**	-997.3218878	0.00	53.26%
**2-2**	-997.3206989	0.75	15.12%
**2-3**	-997.3204524	0.90	11.65%
**2-4**	-997.3196732	1.39	5.10%
**2-5**	-997.3193713	1.58	3.71%
**2-6**	-997.3193429	1.60	3.60%
**2-7**	-997.3187155	1.99	1.85%
**2-8**	-997.3186885	2.01	1.80%
**2-9**	-997.3185741	2.08	1.59%
**2-10**	-997.3183157	2.24	1.21%
**2-11**	-997.3182346	2.29	1.11%
